# Comparative effects of different exercise modalities and doses on lipid metabolism in patients with type 2 diabetes mellitus: a systematic review with Bayesian dose–response network meta-analysis

**DOI:** 10.3389/fendo.2026.1947147

**Published:** 2026-10-07

**Authors:** Jing Liu, Runjie Shi, Wenhui Fu, Ming Zhang

**Affiliations:** 1 School of Wushu and Arts, Nanjing Sport Institute, Nanjing, China; 2 Nanyou Campus, Primary School Division, Xianlin School, High School Affiliated to Nanjing Normal University, Nanjing, China

**Keywords:** Bayesian network meta-analysis, dose-response relationship, exercise, lipid metabolism, type 2 diabetes mellitus

## Abstract

**Objective:**

This review aimed to determine whether the effects of structured exercise on triglycerides (TG) and low-density lipoprotein cholesterol (LDL-C) differed according to exercise modality and weekly training volume in adults with type 2 diabetes mellitus (T2DM).

**Methods:**

Seven electronic databases were searched from inception to 18 December 2025. Randomized controlled trials were eligible when they enrolled adults with T2DM, evaluated a structured exercise intervention, and reported TG or LDL-C outcomes. Comparative effects across exercise categories were then estimated with a Bayesian random-effects network model. Weekly exercise volume was converted to MET-min/week and further examined using model-based dose–response network meta-analysis.

**Results:**

Seventy RCTs comprising 5,317 adults with T2DM met the eligibility criteria. The TG analysis was based on 68 trials and 5,216 participants, whereas the LDL-C analysis included 61 trials and 4,473 participants. In the Bayesian network model, all active exercise modalities were associated with greater reductions in TG and LDL-C than the non-exercise control condition. Among the included modalities, high-intensity interval training (HIIT) produced the most favorable estimate for TG reduction (MD = -0.48 mmol/L, 95% CrI: -0.67, -0.30; SUCRA = 92.53%), while combined aerobic and resistance exercise (CE) showed the highest ranking for LDL-C improvement (MD = -0.46 mmol/L, 95% CrI: -0.63, -0.29; SUCRA = 85.45%). Dose-response modeling indicated that the lipid-lowering effects did not increase in a strictly linear manner across weekly exercise volume. The 95% CrIs remained below zero at approximately 1100–1500 MET-min/week for TG and 950–1500 MET-min/week for LDL-C.

**Conclusions:**

The lipid response to exercise varied according to both training modality and weekly volume. HIIT generated the most favorable estimate for TG, whereas combined exercise showed the leading estimate for LDL-C. The identified dose intervals may provide quantitative reference points for exercise planning, but they should not be treated as fixed prescriptions because certainty of evidence, physical capacity, adherence, treatment background, and safety differ among patients.

**Systematic review registration:**

https://www.crd.york.ac.uk/PROSPERO/view/CRD420261412956, identifier CRD420261412956.

## 1 Introduction

The global burden of diabetes continues to increase. Approximately 537 million adults were living with diabetes in 2021, with projections suggesting an increase to 783 million by 2045 (1). As the affected population continues to grow, the burden of managing metabolic abnormalities and related complications in type 2 diabetes mellitus (T2DM) is increasing. In the pathological progression of T2DM, dyslipidemia is an important metabolic abnormality. Clinically, this lipid disorder commonly involves increased triglyceride (TG) concentrations, lower high-density lipoprotein cholesterol (HDL-C), higher low-density lipoprotein cholesterol (LDL-C), and a greater predominance of small dense LDL particles (2). LDL-C is an important lipid target in atherosclerotic cardiovascular disease (ASCVD) risk management, whereas elevated TG levels usually indicate a greater burden of triglyceride-rich lipoproteins and remnant particles, which may be associated with residual ASCVD risk (3). Compared with T2DM alone, the coexistence of dyslipidemia appears to further increase ASCVD risk in this population. Some studies suggest that this risk may be increased by approximately two-fold (4–6). Therefore, improving dyslipidemia is essential for reducing long-term cardiovascular risk and should be incorporated into the routine management of patients with T2DM.

Exercise is widely incorporated into the management of T2DM because it may support lipid regulation, insulin sensitivity, and inflammatory control. Clinical guidelines also recommend regular exercise as part of the management of dyslipidemia in this population (7–9). Structured programs involving continuous aerobic exercise (CAE), resistance training (RT), HIIT, CE have each been associated with favorable changes in lipid profiles, although their comparative benefits remain uncertain (10–12). Nevertheless, important gaps remain in the current evidence base. Existing syntheses have not fully clarified how different exercise modalities compare with one another or how lipid responses vary across exercise dose. Most conventional meta-analyses have examined individual exercise types separately, which limits direct comparisons across modalities (13). They also lack a comprehensive dose-response framework, which limits their ability to examine potential nonlinear associations between exercise dose and improvements in lipid metabolism. Research specifically addressing resistance-training intensity in relation to lipid outcomes remains limited (14), and other studies have more broadly examined different forms of physical activity (8, 15–17). Although these studies provide preliminary evidence, dose-response evidence remains limited for specifying exercise frequency, intensity, and duration, which are key components of the FITT principle (9, 18). Moreover, previous NMAs in the T2DM field have primarily evaluated exercise-related changes in glycemic outcomes rather than lipid metabolism (19). High-level evidence syntheses remain relatively limited for dyslipidemia, despite its comparable importance in the management of T2DM. The few NMAs that have addressed lipid metabolism have also tended to overlook differences in exercise dose (17, 20, 21). As a result, high-quality evidence remains insufficient to support individualized and precise exercise prescription in clinical practice. This evidence gap limits the translation of exercise findings into individualized lipid-management practice for T2DM (5, 18).

Given these gaps, this study systematically integrated Bayesian network meta-analysis (NMA) with dose-response modeling. Exercise exposure was converted to MET-min/week before dose–response modeling, allowing weekly volume to be examined on a common scale across studies. This study had two objectives. The first was to compare and rank the effects of different exercise modalities on TG and LDL-C in patients with T2DM, thereby informing modality selection. The second was to characterize how lipid responses changed across weekly exercise doses, with the goal of generating quantitative support for individualized exercise planning in lipid management.

## 2 Methods

### 2.1 Registration

The protocol was entered prospectively in PROSPERO under registration number CRD420261412956. Preparation of the review followed the PRISMA 2020 statement and the reporting extension developed for network meta-analysis (22). Because the dataset consisted exclusively of study-level information already available in published reports, institutional ethical review and individual consent were not applicable.

### 2.2 Search strategy

Searches were conducted in PubMed, Web of Science, Embase, the Cochrane Library, China National Knowledge Infrastructure (CNKI), Wanfang, and VIP database. CNKI was included as the principal comprehensive Chinese database, while Wanfang and VIP were searched as complementary sources because their coverage is not completely overlapping, thereby improving retrieval of Chinese-language randomized trials, including studies of traditional Chinese sports. Coverage extended from the earliest date available in each database to 18 December 2025. The search syntax was constructed around terms describing T2DM, exercise or physical activity, and randomized trials. The wording of the search was adjusted for each database using both indexed terms and keywords. Complete search strings are reported in **Supplementary Material 2**.

### 2.3 Eligibility criteria

Eligibility criteria were defined using the PICOS framework. We included RCTs involving adults (≥18 years) with clinically diagnosed T2DM. Participants with stable chronic comorbidities, such as hypertension, dyslipidemia, or coronary heart disease, were considered eligible. Studies were excluded if they involved type 1 or gestational diabetes, pregnancy, acute diabetic complications, severe active infection, diabetic nephropathy, diabetic retinopathy, diabetic foot, or chronic kidney disease. The intervention was a structured exercise program, including six exercise modalities: CAE, RT, HIIT, CE, traditional Chinese sports (TCS), and yoga. To allow sufficient time for physiological adaptation, exercise programs were required to last at least 4 weeks. Control conditions included usual care, health education, waiting-list control, or other management strategies that did not involve structured exercise training. All exercise interventions were considered add-on interventions to the corresponding control condition. Eligible studies were required to report baseline and post-intervention data for at least one of the following outcomes: TG or LDL-C. Studies were also eligible if they provided sufficient information to calculate change values. The study design was restricted to randomized controlled trials (RCTs).

The exclusion criteria were non-randomized studies, duplicate publications, non-T2DM populations, animal studies, reviews or conference abstracts, unclear exercise protocols, interventions lasting less than 4 weeks, and unavailable key outcome data after contacting the authors.

### 2.4 Study selection and data extraction

Candidate reports were reviewed against the prespecified eligibility criteria by JL and WF. The same reviewers independently entered eligible study information into a structured extraction form. Differences in study eligibility or extracted values were resolved through discussion; MZ made the final decision when consensus could not be achieved. Recorded information included author and publication details, country, sample size, participant characteristics, exercise prescription, background treatment, adherence, analysis population, and lipid outcomes. Participant variables comprised age, sex distribution, BMI, and diabetes duration. Intervention variables included exercise category, program length, weekly frequency, and session duration. Information on insulin treatment and changes in medication or diet was also collected where available. Corresponding authors were contacted when reported outcome information was incomplete or required clarification. Inclusion in the quantitative analysis of TG or LDL-C was determined separately according to the usable data available for that outcome.

### 2.5 Data coding and management

Exercise exposure was standardized as weekly metabolic volume (23). For each component of an intervention session, the assigned MET value was multiplied by the number of minutes performed per session and the number of sessions completed each week. Component estimates were then added to produce the total MET-min/week for that intervention arm.

Warm-up, main training, interval recovery, and cool-down were coded separately whenever the intervention contained more than one component. This component-based procedure was used instead of assigning one MET value to the full session. Activity codes and MET values were selected by matching the reported exercise type and intensity to the 2024 Compendium of Physical Activities. The adult compendium was used for studies in which mean participant age was below 60 years, whereas the older-adult compendium was prioritized when mean age was 60 years or above (24, 25). For programs with progressive increases in session length, calculations were based on the average session duration over the full intervention.

For construction of the dose network, calculated weekly volumes were assigned to seven prespecified nodes: 0, 250, 500, 750, 1000, 1250, and 1500 MET-min/week (26, 27). The zero-dose node represented control conditions without structured exercise, while the remaining nodes represented increasing weekly exercise volumes.

### 2.6 Risk of bias assessment

Two reviewers independently applied the revised Cochrane risk-of-bias tool for randomized trials (RoB 2) to each included study (28). Overall judgments were categorized as low risk, some concerns, or high risk of bias. Any unresolved disagreements were adjudicated by a third reviewer.

### 2.7 Credibility assessment

The certainty of each network estimate was judged using the Confidence in Network Meta-Analysis (CINeMA) framework (29). Separate assessments were undertaken for TG and LDL-C, covering comparisons of each active exercise intervention with the control condition and comparisons between active exercise modalities. Judgments across within-study bias, reporting bias, indirectness, imprecision, heterogeneity, and incoherence were integrated to assign an overall certainty rating of high, moderate, low, or very low.

### 2.8 Statistical analysis

#### 2.8.1 Effect size calculation and pairwise meta-analysis

TG and LDL-C were expressed in mmol/L and summarized as mean differences (MDs), with negative values favoring exercise. Change-from-baseline values were used; when change-score SDs were unavailable, they were estimated from baseline and post-intervention SDs assuming a within-participant correlation of 0.50 (30). Other dispersion measures were converted to SDs using established methods. Direct exercise–control comparisons were synthesized using pairwise meta-analysis, and pooled effects were reported as MDs with 95% credible intervals (CrIs). Statistical heterogeneity was assessed using the *I²* statistic. Publication bias was assessed using comparison-adjusted funnel plots and Egger’s regression test (31).

#### 2.8.2 Bayesian NMA

Comparative effects across exercise modalities were estimated using Bayesian random-effects network meta-analysis (NMA) with the gemtc package in R (32). Separate networks were constructed for TG and LDL-C. Posterior estimates were obtained using four Markov chains, with 5,000 iterations discarded as burn-in followed by 20,000 sampling iterations. Convergence was assessed by visual inspection of trace plots. Model fit was evaluated using the deviance information criterion (DIC) and residual deviance, and between-study heterogeneity was summarized using the posterior between-study SD with its 95% CrI. Global inconsistency was examined by comparing the consistency model with the unrelated mean effects (UME) model, with a DIC difference <5 considered to indicate no important disagreement. Where both direct and indirect evidence were available, node-splitting was used to assess local inconsistency, with *p* < 0.05 indicating potential inconsistency (33). Treatment effects were reported as MDs with 95% CrIs, and relative rankings were summarized using the surface under the cumulative ranking curve (SUCRA) (34).

#### 2.8.3 Dose–response network meta-analysis

Weekly exercise dose, expressed as MET-min/week, was incorporated as the exposure variable in a Bayesian random-effects model-based network meta-analysis (MBNMA), with changes in TG and LDL-C as the corresponding outcomes (35). Dose networks were constructed according to the exercise modality and dose assigned to each intervention arm. Before model fitting, network connectivity, transitivity, and consistency were assessed to determine the suitability of the available evidence for indirect comparisons (36–38), with detailed results provided in **Supplementary Material 8**.

The distribution of observed lipid responses across weekly exercise doses was first examined graphically. Four candidate dose–response functions were then fitted: Emax, restricted cubic spline, quadratic, and nonmonotonic models (39). Model selection was based on the overall goodness of fit, considering the DIC, effective number of parameters (pD), residual deviance, between-study SD, and deviance plots. The selected model was used to estimate posterior MDs and 95% CrIs across the dose range. A dose was considered to provide evidence of improvement when its 95% CrI excluded zero, and these estimates were used to identify the dose intervals associated with significant reductions in TG or LDL-C. Posterior ranking probabilities were also estimated for different exercise modality–dose combinations (40). All MBNMA analyses were performed in R version 4.5.2 using the MBNMAdose package.

#### 2.8.4 Additional analyses

Network meta-regression was conducted to explore potential effect modification by study-level characteristics, including sample size, publication year, intervention frequency and duration, proportion of male participants, BMI, age, and baseline lipid concentration. All covariates were analyzed as continuous variables. The robustness of the primary findings was assessed in two sensitivity analyses by excluding studies at high risk of bias and, separately, studies with at least one intervention arm containing fewer than 10 participants. Effect estimates and intervention rankings from these restricted datasets were compared with those from the primary analyses. Additional exploratory subgroup NMAs were conducted according to study-level mean age (<60 vs. ≥60 years) and BMI (<30 vs. ≥30 kg/m^2^) to provide clinically interpretable assessments of potential heterogeneity.

## 3 Results

### 3.1 Literature search results and basic characteristics of included studies

**Figure 1** summarizes the study selection process. The database search yielded 13,820 records, of which 8,678 remained for title and abstract screening after duplicate removal. A total of 172 articles underwent full-text review, and 70 RCTs were retained for quantitative synthesis. Of these, 68 trials contributed TG data and 61 contributed LDL-C data for the NMA. The 70 RCTs included 5,317 participants. CAE was evaluated in 41 studies, followed by RT in 18, TCS in 16, CE in 15, HIIT in 10, and yoga in 4. The basic characteristics and intervention details of the included studies are provided in **Supplementary Material 3**.

**Figure 1:**
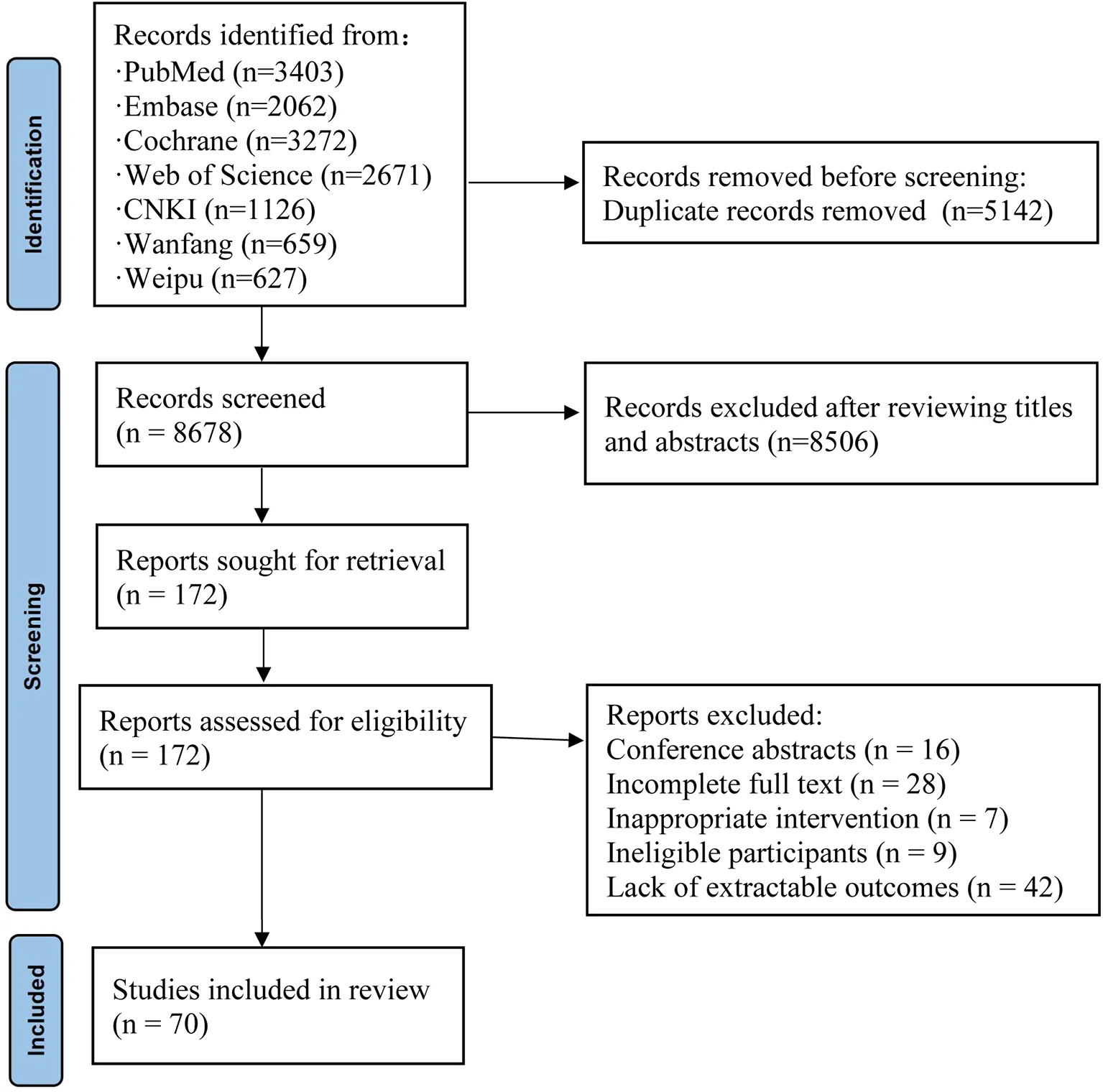
Flow diagram of the literature search and study selection process.

### 3.2 Risk of bias and evidence assessment

Of the 70 trials, 11 were rated as low risk of bias, 54 as having some concerns, and 5 as high risk. Detailed domain-level judgments are provided in **Supplementary Material 4**. Evidence credibility was generally low to moderate for both TG and LDL-C. Moderate credibility was observed for RT in TG and for CE in LDL-C, whereas most other comparisons were rated as low. The main reasons for downgrading were within-study bias, indirectness, and heterogeneity, as detailed in **Supplementary Material 5**.

### 3.3 Pairwise meta-analyses

The results of the pairwise meta-analyses are presented in **Supplementary Material 6**. Direct evidence showed significant TG reductions for CAE, CE, HIIT, RT, and TCS. Yoga also favored lower TG, although its CrI included zero. For LDL-C, significant reductions were observed for CAE, CE, RT, and TCS. HIIT and yoga also showed a tendency toward lower LDL-C, but the credible intervals crossed 0, and these findings should therefore be interpreted with caution. Regarding publication bias, the TG funnel plot showed no obvious asymmetry, and Egger’s tests were non-significant for both TG (*p* = 0.2760) and LDL-C (*p* = 0.0722).

### 3.4 NMA

The TG network included 68 RCTs and 5,216 participants (**Figure 2a**). All six exercise modalities produced greater reductions than CG (**Table 1**). HIIT showed the largest estimated reduction in TG (MD = -0.48 mmol/L, 95% CrI: -0.67, -0.30), followed by yoga (MD = -0.35 mmol/L, 95% CrI: -0.64, -0.05), TCS (MD = -0.34 mmol/L, 95% CrI: -0.48, -0.20), CE (MD = -0.30 mmol/L, 95% CrI: -0.46, -0.14), RT (MD = -0.28 mmol/L, 95% CrI: -0.44, -0.12), and CAE (MD = -0.27 mmol/L, 95% CrI: -0.37, -0.17). The cumulative ranking based on SUCRA showed that HIIT ranked highest for TG reduction (SUCRA = 92.53%), followed by TCS (62.25%), yoga (61.05%), CE (50.79%), RT (44.18%), CAE (39.00%), and CG (0.20%) (**Figure 2c**).

**Figure 2:**
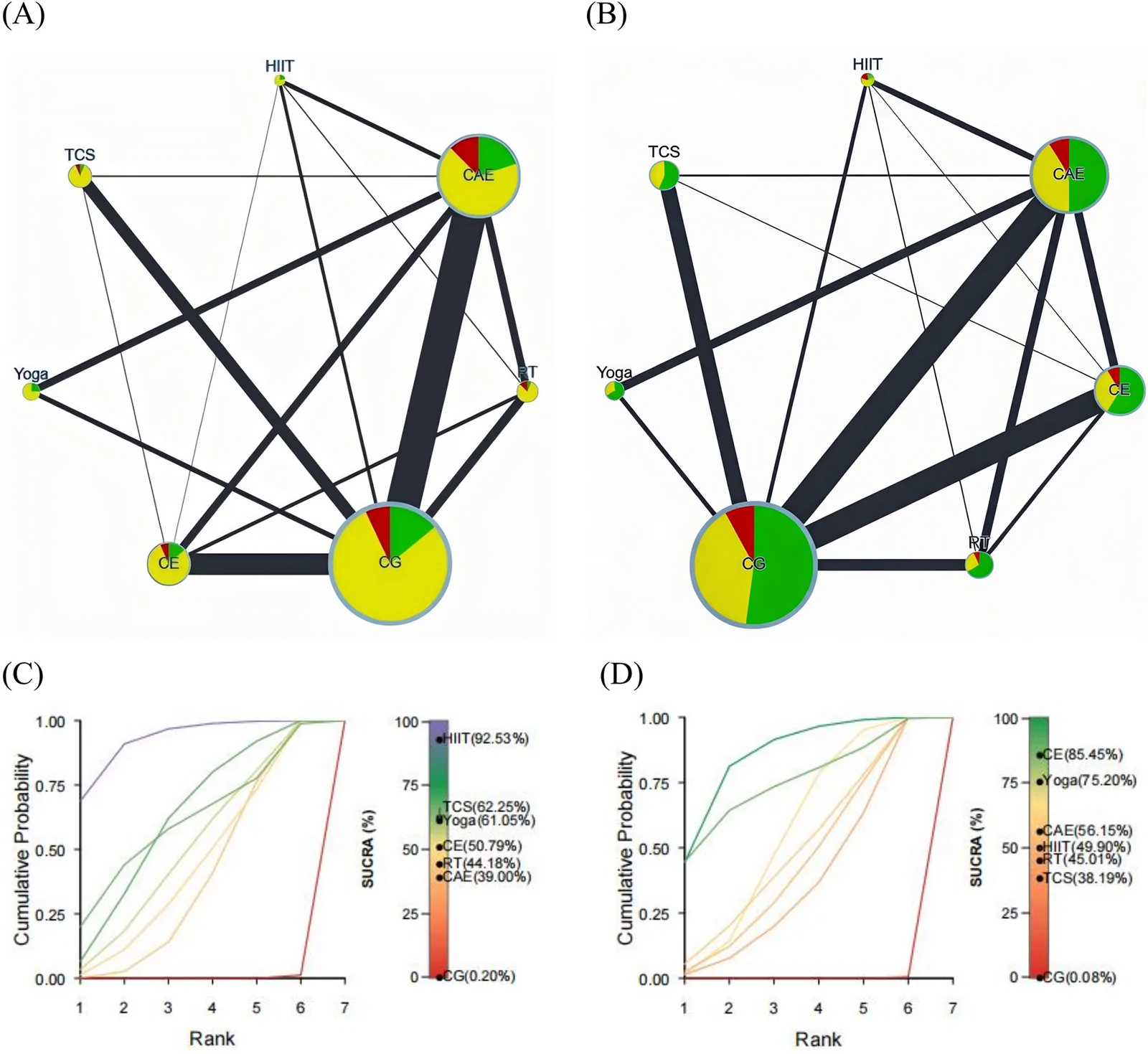
Network plots and SUCRA ranking plots for the effects of different exercise interventions on TG and LDL-C. Panels **(A, B)** show the network plots for TG and LDL-C, respectively; node size represents sample size, and edge thickness represents the number of direct comparisons. Panels **(C, D)** show the corresponding SUCRA rankings, with higher values indicating higher rankings. SUCRA, surface under the cumulative ranking curve.

**Table 1:** Comparison of the effects of different exercise interventions on lipid profile outcomes.

Outcomes	Comparison						
TG	CAE						
	0.03 (-0.14, 0.20)	CE					
	0.21 (0.03, 0.39)	0.18 (-0.06, 0.41)	HIIT				
	0.01 (-0.15, 0.17)	-0.02 (-0.23, 0.19)	-0.20 (-0.41, 0.02)	RT			
	0.07 (-0.10, 0.23)	0.04 (-0.17, 0.24)	-0.14 (-0.37, 0.09)	0.06 (-0.15, 0.26)	TCS		
	0.07 (-0.23, 0.37)	0.04 (-0.29, 0.37)	-0.13 (-0.48, 0.21)	0.07 (-0.27, 0.39)	0.01 (-0.32, 0.34)	Yoga	
	**-0.27 (-0.37, -0.17)**	**-0.30 (-0.46, -0.14)**	**-0.48 (-0.67, -0.30)**	**-0.28 (-0.44, -0.12)**	**-0.34 (-0.48, -0.20)**	**-0.35 (-0.64, -0.05)**	CG
LDL-C	CAE						
	0.11 (-0.07, 0.30)	CE					
	-0.02 (-0.21, 0.16)	-0.13 (-0.37, 0.11)	HIIT				
	-0.03 (-0.20, 0.13)	-0.14 (-0.37, 0.07)	-0.01 (-0.24, 0.21)	RT			
	-0.06 (-0.23, 0.11)	-0.17 (-0.39, 0.04)	-0.04 (-0.28, 0.19)	-0.02 (-0.24, 0.19)	TCS		
	0.10 (-0.23, 0.42)	-0.01 (-0.39, 0.35)	0.12 (-0.25, 0.48)	0.13 (-0.23, 0.48)	0.16 (-0.20, 0.52)	Yoga	
	**-0.35 (-0.46, -0.24)**	**-0.46 (-0.63, -0.29)**	**-0.33 (-0.52, -0.14)**	**-0.31 (-0.48, -0.15)**	**-0.29 (-0.43, -0.15)**	**-0.44 (-0.77, -0.12)**	CG

Values are MDs with 95% CrIs estimated from the Bayesian random-effects NMA. Negative values indicate greater reductions in TG or LDL-C, and bold values indicate that the 95% CrI did not include zero. MD, mean difference; CrI, credible interval; TG, triglyceride; LDL-C, low-density lipoprotein cholesterol.

The LDL-C network comprised 61 RCTs with 4,473 participants (**Figure 2b**). Each active exercise modality was favored over CG (**Table 1**). Compared with the CG, all exercise interventions significantly reduced LDL-C levels. CE showed the largest estimated reduction in LDL-C (MD = -0.46 mmol/L, 95% CrI: -0.63, -0.29), followed by yoga (MD = -0.44 mmol/L, 95% CrI: -0.77, -0.12), CAE (MD = -0.35 mmol/L, 95% CrI: -0.46, -0.24), HIIT (MD = -0.33 mmol/L, 95% CrI: -0.52, -0.14), RT (MD = -0.31 mmol/L, 95% CrI: -0.48, -0.15), and TCS (MD = -0.29 mmol/L, 95% CrI: -0.43, -0.15). The cumulative ranking based on SUCRA showed that CE ranked highest for LDL-C reduction (SUCRA = 85.45%), followed by yoga (75.20%), CAE (56.15%), HIIT (49.90%), RT (45.01%), TCS (38.19%), and CG (0.08%) (**Figure 2d**).

### 3.5 Dose-response network meta-analysis

A nonlinear dose-response relationship was observed between weekly exercise dose and reductions in TG levels (**Figure 3a**). Based on the predicted effects from the final model, and using a 95% CrI that did not cross 0 as the criterion for statistical significance, the weekly exercise dose range associated with significant TG improvement was approximately 1100–1500 MET-min/week. The dose-response relationships for different exercise modalities are shown in **Figure 3b**. Ranking probability analysis indicated that HIIT (1000 MET-min/week), HIIT (890 MET-min/week), and TCS (1500 MET-min/week) were more likely to produce larger reductions in TG (**Supplementary Table S18**).

**Figure 3:**
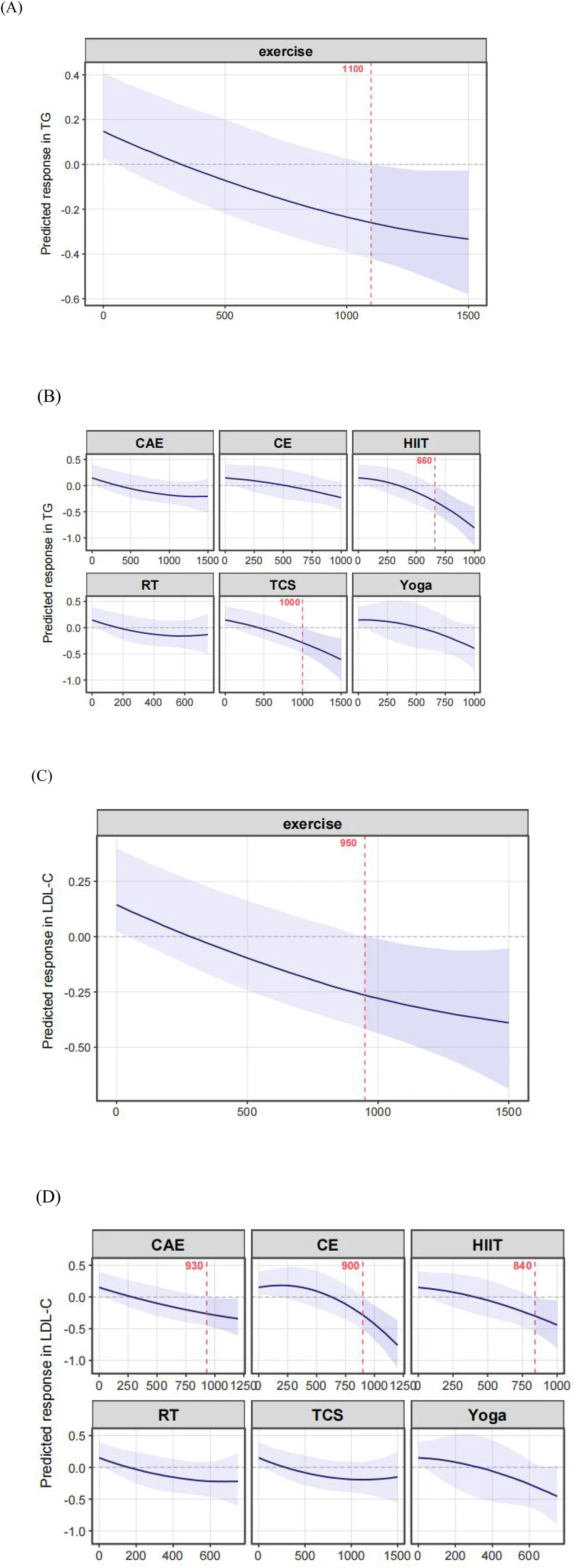
Dose–response relationships between weekly exercise dose and improvements in TG and LDL-C. Dose–response curves were estimated using Bayesian random-effects MBNMA. Panels **(A, C)** show the overall curves for TG and LDL-C, and Panels **(B, D)** show modality-specific curves. Negative estimates indicate greater lipid reductions; shaded areas represent 95% CrIs, and red dashed lines indicate the estimated dose at which the 95% CrI first excluded zero. MBNMA, model-based network meta-analysis; CrI, credible interval.

For LDL-C, the estimated response curve also suggested a nonlinear pattern across weekly exercise doses (**Figure 3c**). Using a 95% CrI that did not cross 0 as the criterion for statistical significance, the weekly exercise dose range associated with significant LDL-C improvement was approximately 950–1500 MET-min/week. The dose-response relationships for different exercise modalities are shown in **Figure 3d**. Ranking probability analysis indicated that CE (1200 MET-min/week), CE (1100 MET-min/week), and HIIT (1000 MET-min/week) were more likely to produce larger improvements in LDL-C (**Supplementary Table S19**).

### 3.6 Additional analyses

Network meta-regression suggested that baseline TG levels may influence the relative improvement in TG across different exercise modalities in patients with T2DM. Baseline LDL-C levels and BMI may influence the relative improvement in LDL-C. Other included covariates did not show significant moderating effects on TG or LDL-C.

Sensitivity analyses showed that the ranking of exercise interventions and the statistical significance of the effects did not change substantially. These findings suggest that the main results were relatively robust. Detailed results are provided in **Supplementary Materials 7.6**, **7.7**. Subgroup analyses showed that all six exercise modalities significantly reduced both TG and LDL-C in studies with a mean age <60 years. In studies with a mean age ≥60 years, HIIT and TCS remained significant for TG and CE for LDL-C. For BMI, CAE, CE, HIIT, RT, and TCS significantly reduced both outcomes in studies with a mean BMI <30 kg/m², whereas in studies with a mean BMI ≥30 kg/m^2^. HIIT remained significant for TG and CAE, CE, HIIT, and RT for LDL-C (**Supplementary Figure S7**).

## 4 Discussion

This review extends previous evidence by combining Bayesian NMA with dose–response modeling and by standardizing exercise dose as MET-min/week. This framework enabled simultaneous comparison of six exercise modalities and their dose-related associations with TG and LDL-C in patients with T2DM. The main findings were as follows. First, HIIT showed a relatively greater potential benefit for reducing TG. Second, CE appeared to have a more favorable effect on LDL-C reduction. Third, unlike the preliminary findings from conventional meta-analysis, the NMA showed that all six exercise interventions significantly improved TG and LDL-C levels in patients with T2DM. Notably, yoga ranked among the leading interventions for both outcomes. Dose–response modeling also indicated that lipid benefits varied across weekly exercise doses rather than following a simple linear pattern. The main findings and proposed mechanisms are summarized in **Figure 4**.

**Figure 4:**
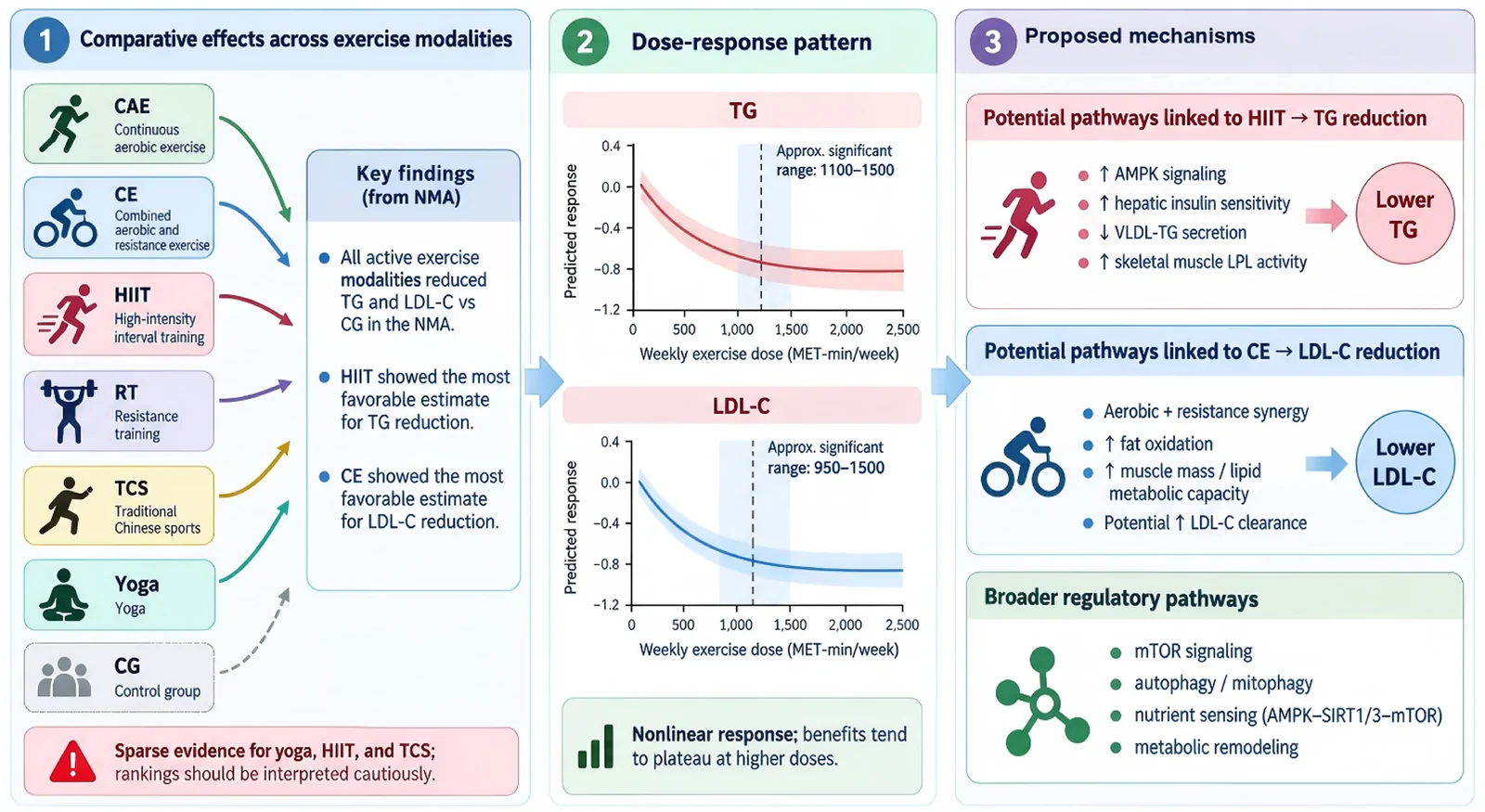
Schematic diagram of the main findings and proposed mechanisms.

The results showed that HIIT had the largest estimated effect on TG reduction (MD = -0.48 mmol/L, 95% CrI: -0.67, -0.30), with a SUCRA value of 92.53%. This finding differs from previous studies on exercise interventions for lipid metabolism in patients with T2DM. Most previous meta-analyses and clinical studies have suggested that CAE is the preferred exercise modality for improving lipid profiles in patients with T2DM, with greater effects on TG and LDL-C reduction than HIIT or RT (12, 13). This discrepancy may be explained by several factors. First, from a methodological perspective, the network structure incorporated both head-to-head and indirect evidence, enabling all six exercise modalities to be compared within the same model. This would not be possible using conventional pairwise meta-analysis alone (41, 42). Second, we standardized exercise dose using a unified metric and excluded studies with poorly defined intervention protocols or short intervention durations (<4 weeks). These design features may have allowed the effects of HIIT to be more clearly captured. Mechanistically, it is important to distinguish the acute lipolytic response from chronic metabolic adaptations. However, the following interpretations are biologically plausible hypotheses derived from the broader literature and cannot be directly inferred from this study level meta-analysis. The acute response is largely mediated by catecholamine-stimulated HSL activation (43), which is insufficient to explain sustained fasting TG reductions. The observed improvement more likely reflects coordinated chronic adaptations. As a candidate mechanism, long-term HIIT has been proposed to activate AMPK signaling and improve hepatic insulin sensitivity (44), potentially suppressing hepatic VLDL-TG secretion, and has been suggested to upregulate skeletal muscle LPL activity (45), accelerating clearance of TG-rich lipoproteins (45, 46). These pathways remain hypothetical, the present meta-analysis directly supports only the HIIT-TG association, and molecular verification requires dedicated mechanistic studies. The short-term lipolytic effect may play a supportive role mainly through accumulated energy expenditure and body composition improvements (47). Beyond the AMPK-LPL-VLDL axis, emerging evidence implicates mTOR signaling, autophagy, and nutrient-sensing pathways in exercise-induced metabolic regulation. mTORC1 integrates nutrient availability and energy status to regulate protein synthesis, lipid metabolism, and mitochondrial function; recent studies highlight interactions between exercise, mTOR activity, insulin sensitivity, and metabolic remodeling (48, 49). Acute exercise transiently suppresses mTORC1, while post-exercise recovery promotes reactivation; chronic training may modulate mTOR signaling sensitivity. Exercise also induces mitophagy in skeletal muscle and liver, clearing damaged organelles and potentially improving insulin sensitivity (50, 51). The mTOR–autophagy interplay is bidirectional: mTORC1 inhibits autophagy under nutrient-replete conditions, while exercise-induced AMPK activation suppresses mTORC1 and promotes autophagy via ULK1. Nutrient-sensing pathways involving sirtuins (SIRT1/3), AMPK, and mTOR collectively regulate metabolic gene expression. These candidate mechanisms may partly explain modality-specific lipid responses, but the present meta-analysis did not measure mTOR activity or autophagy flux; they remain hypotheses requiring molecular verification. By contrast, although CAE also increases energy expenditure, its effects on reversing hepatic insulin resistance and upregulating skeletal muscle LPL activity may be less rapid and less pronounced than those induced by the intense metabolic stress of HIIT (52). This may partly explain why the reduction in TG associated with CAE was smaller than that observed with HIIT.

For LDL-C, CE showed the largest estimated reduction (MD = -0.46 mmol/L, 95% CrI: -0.63, -0.29; SUCRA = 85.45%). This finding is consistent with several high-quality clinical trials showing that combined training has favorable effects on lipid profiles, including LDL-C (53). Our ranking results partly diverged from a previous NMA, which favored aerobic exercise for TG reduction but did not identify combined aerobic and resistance training as the top-ranked strategy for LDL-C (54). This inconsistency may be explained by differences in study selection criteria, the number of included studies, and the definitions of exercise modalities. Our study included more recently published RCTs that examined specific exercise modalities, which may provide more refined evidence. Mechanistically, CE’s advantage may reflect potential synergies, though this meta-analysis cannot verify the underlying mechanisms. The aerobic component is generally considered to promote fat oxidation (55); the resistance component may increase muscle mass, enhancing lipid metabolic capacity and potentially LPL activity and hepatic LDL-C clearance (56–58). These interpretations remain hypothetical and require confirmation through studies incorporating body composition and molecular measurements.

Another noteworthy finding was that the NMA results differed from the preliminary findings of the conventional pairwise meta-analysis. Yoga showed significant favorable effects on both TG and LDL-C, ranking second for LDL-C (SUCRA = 75.20%). These require particularly cautious interpretation: yoga was supported by only 4 TG trials and 3 LDL-C trials with wide credible intervals. Among six modalities, yoga had the smallest evidence base; HIIT (10 trials) and TCS (16 trials) also had substantially smaller bases than CAE (41 trials). Smaller evidence bases mean SUCRA rankings may be less stable and more susceptible to individual-study influence. Yoga’s benefits may involve stress regulation and improved insulin sensitivity, making it feasible for patients unable to tolerate higher-intensity exercise, but its lipid-lowering effects require confirmation in well-powered RCTs. By contrast, NMA can incorporate indirect comparative evidence and expand the information base, which may improve the ability to detect effects for interventions with smaller evidence bases. The potential benefits of yoga may not primarily result from high energy expenditure, but rather from a combination of mechanisms. First, yoga may support stress regulation. Through breathing control and meditation, yoga can help reduce stress-related hormones such as cortisol, thereby indirectly improving stress-related insulin resistance and dyslipidemia (59, 60). Second, yoga may improve insulin sensitivity (61). Sustained postural practice and muscle engagement may enhance insulin signaling in peripheral tissues. Therefore, for patients with T2DM who have difficulty participating in higher-intensity exercise because of limited physical capacity or personal preference, yoga may be a feasible option (62, 63). However, its lipid-lowering effects still need to be confirmed in more high-quality studies.

In addition, dose–response modeling suggested that the lipid response to exercise did not increase linearly with weekly dose. This finding differs from earlier evidence. Based on limited observations, Aune (16) suggested a weekly exercise dose of at least 1500 MET-min/week and tended to support a simple linear interpretation, in which higher exercise doses were assumed to produce greater improvements in lipid metabolism (16). Similarly, the dose-based study of resistance training by Fan (14) was limited to group-level comparisons. It therefore reached only a relatively broad conclusion that moderate-intensity RT was superior to low-intensity RT, without characterizing dose ranges associated with statistically significant lipid improvement (14). Using the 2024 Compendium of Physical Activities, we standardized the intensity coding for six exercise modalities and further classified exercise dose into seven levels. This approach helped address the problems of inconsistent dose grouping and limited data comparability in previous studies. After comparing multiple model-fit indicators, we selected the quadratic model with the best overall fit. This model allowed us to better capture the dynamic association between exercise dose and response. We found that improvements in TG and LDL-C were more likely to reach statistical significance once the weekly exercise dose reached a certain level. At higher dose ranges, however, the curve tended to flatten. This suggests that further increases in exercise dose may provide only limited marginal benefits (64–66). The observed plateau may reflect several physiological constraints: first, the maximal capacity of LPL-mediated TG clearance and hepatic LDL receptor expression may be reached beyond a certain exercise volume, leaving limited room for additional improvement (23); second, compensatory increases in appetite and energy intake at higher exercise volumes may offset the additional energy expenditure, attenuating the net metabolic benefit; third, excessive training volume could induce overtraining-related elevations in cortisol and systemic low-grade inflammation (67), which may counteract the beneficial lipid effects. These candidate mechanisms remain speculative and require dedicated dose-escalation studies with controlled energy intake monitoring.

The overall dose–response analysis showed that the weekly exercise dose ranges associated with statistically significant improvements in TG and LDL-C were approximately 1100–1500 and 950–1500 MET-min/week, respectively. When modality–dose combinations were ranked, HIIT at approximately 890–1000 MET-min/week appeared more favorable for TG reduction, whereas CE at approximately 1100–1200 MET-min/week showed higher probabilities of LDL-C reduction. These findings provide quantitative evidence to support more precise clinical exercise prescription and may help avoid the misconception that simply increasing exercise volume will necessarily lead to greater benefits.

It should be noted that the robustness of our findings was supported by several analyses. First, Egger’s tests did not indicate clear evidence of publication bias, which provides some reassurance regarding the stability of the findings. Second, between-study heterogeneity was accounted for by using Bayesian random-effects models. In addition, sensitivity analyses showed that the ranking of exercise interventions and the statistical significance of the effects did not change substantially after excluding studies with a high risk of bias or small sample sizes. These results support the robustness of the main findings. To further explore potential sources of between-study heterogeneity, we conducted network meta-regression analyses. For TG, only baseline TG level was identified as a significant source of heterogeneity (*β* = -0.18, 95% CrI: -0.32, -0.03). This may reflect greater room for lipid improvement among participants with poorer baseline lipid status, although this interpretation should be tested in future individual participant data analyses (8). From a mechanistic perspective, the negative regression coefficient suggests that individuals with higher baseline TG exhibit greater exercise-induced reductions. This may be because patients with elevated baseline TG often have more severe hepatic insulin resistance and greater VLDL-TG overproduction (68); exercise-induced AMPK activation and improvements in hepatic insulin sensitivity may more effectively suppress this overproduction in the context of a dysregulated baseline (69), producing a larger relative improvement. In contrast, individuals with near-normal baseline TG have less metabolic dysfunction to correct, resulting in a smaller observed effect.

For LDL-C, the results suggested a more complex pattern of effect modification. First, the effect of baseline LDL-C level was consistent with that observed for TG (*β* = -0.23, 95% CrI: -0.41, -0.06). Second, BMI showed a significant positive moderating effect (*β* = 0.21, 95% CrI: 0.02, 0.41). The positive regression coefficient indicates that, as BMI increased, the reduction in LDL-C after exercise intervention tended to become smaller. This finding may suggest that, for patients with T2DM and higher BMI, improving LDL-C through the exercise modalities included in this study may be more challenging. More complex mechanisms of metabolic resistance may be involved (70, 71). Mechanistically, the attenuated LDL-C response in higher-BMI participants may be explained by several interrelated factors: higher BMI is associated with greater insulin resistance and more severe hepatic steatosis, both of which can blunt exercise-induced upregulation of hepatic LDL receptor expression; higher-BMI patients are also more likely to be on statin therapy at baseline, which may already lower LDL-C substantially and reduce the marginal benefit of add-on exercise (72); additionally, greater adiposity is associated with higher systemic inflammation and reduced cholesterol efflux capacity, which could further attenuate the lipid-lowering response to exercise. These candidate mechanisms require verification in studies with detailed body composition and medication data.

Our findings have important public health and clinical implications for lipid metabolism management in patients with T2DM. The central implication is that exercise interventions should move away from a one-size-fits-all approach and toward individualized strategies based on specific lipid metabolism targets. Because TG and LDL-C may differ in their responses to exercise modality and dose, clinical and community-based practice should avoid using a single standardized exercise program for all patients (5, 73). Instead, more targeted exercise intervention strategies should be developed according to the lipid outcome that requires priority management. To ensure feasibility and long-term adherence, we recommend a stepwise and progressive implementation pathway. In the initial phase, low-barrier exercise should be used to help patients reach the minimum effective dose while ensuring exercise safety. In the attainment phase, weekly exercise dose should be gradually titrated and maintained within the target range associated with optimal benefit by steadily increasing exercise frequency and duration. In the maintenance phase, the focus should shift to sustaining long-term exercise behavior and maintaining a relatively stable and feasible exercise load, so that improvements in lipid metabolism can be preserved. This process should be accompanied by dynamic monitoring and feedback. Regular assessment of lipid levels, including short-term evaluation of TG and longer-term evaluation of LDL-C, exercise adherence, and adverse events should be used to guide ongoing adjustments to the exercise prescription (74). However, it must be emphasized that, given the moderate-to-low certainty of the evidence and the potential risk of bias in this study, the specific dose ranges described above should be regarded as guidance ranges rather than absolute or precise prescriptions. When translating these findings into clinical practice and public health strategies, individual differences should be fully considered. Real-world effectiveness and safety should also be monitored simultaneously to ensure that these evidence-based recommendations can be implemented safely and effectively across diverse populations.

This review has strengths including combined Bayesian NMA with dose-response modeling, sensitivity analyses, and meta-regression. However, several limitations apply. First, confounding by background treatment and metabolic status. Most participants used glucose-lowering medications; many also received lipid-lowering therapy (statins, fibrates), dietary interventions, or combined lifestyle modifications. Inconsistent reporting precluded robust adjustment. Lipid-lowering medications and diet can independently reduce lipids, and baseline metabolic status may moderate exercise responses; the study-level design could not fully disentangle these confounders. Future studies should systematically report and adjust for medication use (type, dose, changes), lipid-lowering therapy, diet, and baseline metabolic parameters, ideally via individual participant data (IPD) meta-analysis. Second, limited subgroup analyses, sensitivity analyses and exploratory subgroups by age (<60 vs ≥60) and BMI (<30 vs ≥30) were conducted but limited by study-level design and small subgroup sizes. Subgroups by medication use, diabetes duration, baseline level, and exercise prescription characteristics could provide deeper clinical insights but were precluded by inconsistent reporting. Future IPD meta-analyses or large prospective studies with standardized reporting are needed to identify high-benefit subgroups. Third, limited evidence for certain modalities, as noted, yoga, HIIT, and TCS had substantially smaller evidence bases than CAE, reducing estimate precision and ranking stability. Well-powered RCTs for these modalities with standardized lipid assessment are needed. Overall, this study provides comprehensive evidence for exercise-based lipid management in T2DM and direction for precision exercise prescription research.

## 5 Conclusions

This Bayesian NMA with dose-response modeling compared six exercise modalities for lipid management in patients with T2DM. Three key findings emerged. First, HIIT showed the most favorable estimate for TG reduction, whereas CE ranked highest for LDL-C improvement. Second, lipid responses followed nonlinear dose-response patterns, with significant improvements observed at approximately 1100–1500 MET-min/week for TG and 950–1500 MET-min/week for LDL-C. Third, baseline lipid levels and BMI may influence exercise responsiveness. Overall, these findings support more lipid-target-specific and individualized exercise planning, although the moderate-to-low certainty of the evidence warrants confirmation in well-powered RCTs.

## Data Availability

The original contributions presented in the study are included in the article/**Supplementary Material**. Further inquiries can be directed to the corresponding author.

## References

[1] GBD 2021 Diabetes Collaborators . Global, regional, and national burden of diabetes from 1990 to 2021, with projections of prevalence to 2050: a systematic analysis for the Global Burden of Disease Study 2021. Lancet. (2023) 402:203–34. doi: 10.1016/s0140-6736(23)01301-6 37356446 PMC10364581

[2] Yuge H Okada H Hamaguchi M Kurogi K Murata H Ito M . Triglycerides/HDL cholesterol ratio and type 2 diabetes incidence: Panasonic Cohort Study 10. Cardiovasc Diabetol. (2023) 22:308. doi: 10.1186/s12933-023-02046-5 37940952 PMC10634002

[3] Xiao Y Yu B Chao C Wang S Hu D Wu C . Chinese expert consensus on blood lipid management in patients with diabetes (2024 edition). J Transl Int Med. (2024) 12:325–43. doi: 10.2478/jtim-2024-0014 PMC11444477 39360162

[4] Alsaeedi AO . The prevalence of obesity and the associated risk factors among type II diabetic patients attending Al-Aziziyah Al-Sharqiah Primary Health Care Center in Makkah Al-Mukarramah, 2018. Am J Med Sci Med. (2019) 7:80–8. doi: 10.12691/ajmsm-7-3-6

[5] Marx N Federici M Schütt K Müller-Wieland D Ajjan RA Antunes MJ . 2023 ESC Guidelines for the management of cardiovascular disease in patients with diabetes. Eur Heart J. (2023) 44:4043–140. doi: 10.1093/eurheartj/ehad192 37622663

[6] Chait A Eckel RH Vrablik M Zambon A . Lipid-lowering in diabetes: an update. Atherosclerosis. (2024) 394:117313. doi: 10.1016/j.atherosclerosis.2023.117313 37945448

[7] Elsayed NA Aleppo G Aroda VR Bannuru RR Brown FM Bruemmer D . Facilitating positive health behaviors and well-being to improve health outcomes: Standards of Care in Diabetes—2023. Diabetes Care. (2023) 46:S68–96. doi: 10.2337/dc23-S005 36507648 PMC9810478

[8] Smart NA Downes D van der Touw T Hada S Dieberg G Pearson MJ . The effect of exercise training on blood lipids: a systematic review and meta-analysis. Sports Med. (2025) 55:67–78. doi: 10.1007/s40279-024-02115-z 39331324 PMC11787149

[9] Kanaley JA Colberg SR Corcoran MH Malin SK Rodriguez NR Crespo CJ . Exercise/physical activity in individuals with type 2 diabetes: a consensus statement from the American College of Sports Medicine. Med Sci Sports Exerc. (2022) 54:353–68. doi: 10.1249/mss.0000000000002800 35029593 PMC8802999

[10] Francois ME Little JP . Effectiveness and safety of high-intensity interval training in patients with type 2 diabetes. Diabetes Spectr. (2015) 28:39–44. doi: 10.2337/diaspect.28.1.39 25717277 PMC4334091

[11] Yang Z Scott CA Mao C Tang J Farmer AJ . Resistance exercise versus aerobic exercise for type 2 diabetes: a systematic review and meta-analysis. Sports Med. (2014) 44:487–99. doi: 10.1007/s40279-013-0128-8 24297743

[12] Schwingshackl L Missbach B Dias S Konig J Hoffmann G . Impact of different training modalities on glycaemic control and blood lipids in patients with type 2 diabetes: a systematic review and network meta-analysis. Diabetologia. (2014) 57:1789–97. doi: 10.1007/s00125-014-3303-z 24996616

[13] Wood G Taylor E Ng V Murrell A Patil A van der Touw T . Estimating the effect of aerobic exercise training on novel lipid biomarkers: a systematic review and multivariate meta-analysis of randomized controlled trials. Sports Med. (2023) 53:871–86. doi: 10.1007/s40279-023-01817-0 36862340 PMC10036419

[14] Fan T Lin M-H Kim K . Intensity differences of resistance training for type 2 diabetic patients: a systematic review and meta-analysis. Healthcare (Basel). (2023) 11:440. doi: 10.3390/healthcare11030440 36767015 PMC9914423

[15] Li J Zhang W Guo Q Liu X Zhang Q Dong R . Duration of exercise as a key determinant of improvement in insulin sensitivity in type 2 diabetes patients. Tohoku J Exp Med. (2012) 227:289–96. doi: 10.1620/tjem.227.289 22850594

[16] Aune D Norat T Leitzmann M Tonstad S Vatten LJ . Physical activity and the risk of type 2 diabetes: a systematic review and dose–response meta-analysis. Eur J Epidemiol. (2015) 30:529–42. doi: 10.1007/s10654-015-0056-z 26092138

[17] Michielsen M Yagiz J Hanssens M Claes J Geuns L Gojevic T . The effect of exercise characteristics on HbA1c and other cardiovascular risk factors in adults with type 2 diabetes: a systematic review and meta-analysis of randomised controlled trials. Cardiovasc Diabetol. (2025) 25:30. doi: 10.1186/s12933-025-03048-1 41457265 PMC12859889

[18] Collins KA Ross LM Slentz CA Huffman KM Kraus WE . Differential effects of amount, intensity, and mode of exercise training on insulin sensitivity and glucose homeostasis: a narrative review. Sports Med - Open. (2022) 8:90. doi: 10.1186/s40798-022-00480-5 35834023 PMC9283590

[19] Liu H Wang W Sun J . Bayesian network meta-analysis of the impact of exercise therapy on blood glucose in type 2 diabetes patients. Front Endocrinol. (2025) 16:1658739. doi: 10.3389/fendo.2025.1658739 41255539 PMC12620225

[20] Hou L Wang Q Pan B Li R Li Y He J . Exercise modalities for type 2 diabetes: a systematic review and network meta-analysis of randomized trials. Diabetes Metab Res Rev. (2023) 39:e3591. doi: 10.1002/dmrr.3591 36423199

[21] Garcia SP Cureau FV Iorra FQ Bottino LG LE RCM Leivas G . Effects of exercise training and physical activity advice on HbA1c in people with type 2 diabetes: a network meta-analysis of randomized controlled trials. Diabetes Res Clin Pract. (2025) 221:112027. doi: 10.1016/j.diabres.2025.112027 39904457

[22] Hutton B Catalá-López F Moher D . The PRISMA statement extension for systematic reviews incorporating network meta-analysis: PRISMA-NMA. Med Clínica (English Edition). (2016) 147:262–6. doi: 10.1016/j.medcle.2016.10.003 27040178

[23] Ding H Jiang L Seo H Chun B . Optimal exercise modalities and doses for improving pro-atherogenic lipid profiles in patients with metabolic syndrome: a systematic review with pairwise, network, and dose-response meta-analyses. BMC Med. (2025) 23:691. doi: 10.1186/s12916-025-04495-z 41444893 PMC12729105

[24] Herrmann SD Willis EA Ainsworth BE Barreira TV Hastert M Kracht CL . 2024 Adult Compendium of Physical Activities: A third update of the energy costs of human activities. J Sport Health Sci. (2024) 13:6–12. doi: 10.1016/j.jshs.2023.10.010 38242596 PMC10818145

[25] Willis EA Herrmann SD Hastert M Kracht CL Barreira TV Schuna JM Jr. . Older Adult Compendium of Physical Activities: Energy costs of human activities in adults aged 60 and older. J Sport Health Sci. (2024) 13:13–7. doi: 10.1016/j.jshs.2023.10.007 38242593 PMC10818108

[26] Pedder H Dias S Bennetts M Boucher M Welton NJ . Joining the dots: linking disconnected networks of evidence using dose-response model-based network meta-analysis. Med Decis Making. (2021) 41:194–208. doi: 10.1177/0272989x20983315 33448252 PMC7879230

[27] Gallardo-Gómez D Salazar-Martínez E Alfonso-Rosa RM Ramos-Munell J Del Pozo-Cruz J Del Pozo Cruz B . Optimal dose and type of physical activity to improve glycemic control in people diagnosed with type 2 diabetes: a systematic review and meta-analysis. Diabetes Care. (2024) 47:295–303. doi: 10.2337/dc23-0800 38241499

[28] Sterne JAC Savović J Page MJ Elbers RG Blencowe NS Boutron I . RoB 2: a revised tool for assessing risk of bias in randomised trials. BMJ. (2019) 366:l4898. doi: 10.1136/bmj.l4898 31462531

[29] Nikolakopoulou A Higgins JPT Papakonstantinou T Chaimani A Del Giovane C Egger M . CINeMA: An approach for assessing confidence in the results of a network meta-analysis. PloS Med. (2020) 17:e1003082. doi: 10.1371/journal.pmed.1003082 32243458 PMC7122720

[30] Furukawa TA Barbui C Cipriani A Brambilla P Watanabe N . Imputing missing standard deviations in meta-analyses can provide accurate results. J Clin Epidemiol. (2006) 59:7–10. doi: 10.1016/j.jclinepi.2005.06.006 16360555

[31] Lin L Chu H Murad MH Hong C Qu Z Cole SR . Empirical comparison of publication bias tests in meta-analysis. J Gen Intern Med. (2018) 33:1260–7. doi: 10.1007/s11606-018-4425-7 29663281 PMC6082203

[32] Hutton B Salanti G Caldwell DM Chaimani A Schmid CH Cameron C . The PRISMA extension statement for reporting of systematic reviews incorporating network meta-analyses of health care interventions: checklist and explanations. Ann Intern Med. (2015) 162:777–84. doi: 10.7326/m14-2385 26030634

[33] Dias S Welton NJ Sutton AJ Caldwell DM Lu G Ades AE . Evidence synthesis for decision making 4: inconsistency in networks of evidence based on randomized controlled trials. Med Decis Making. (2013) 33:641–56. doi: 10.1177/0272989x12455847 23804508 PMC3704208

[34] Salanti G Ades AE Ioannidis JP . Graphical methods and numerical summaries for presenting results from multiple-treatment meta-analysis: an overview and tutorial. J Clin Epidemiol. (2011) 64:163–71. doi: 10.1016/j.jclinepi.2010.03.016 20688472

[35] Mawdsley D Bennetts M Dias S Boucher M Welton NJ . Model-based network meta-analysis: a framework for evidence synthesis of clinical trial data. CPT Pharmacometrics Syst Pharmacol. (2016) 5:393–401. doi: 10.1002/psp4.12091 27479782 PMC4999602

[36] Belavý DL Kaczorowski S Saueressig T Owen PJ Nikolakopoulou A . How to conduct and report checking transitivity and inconsistency in network-meta-analysis: a narrative review including practical worked examples, code and source data for sports and exercise medicine researchers. BMJ Open Sport Exerc Med. (2024) 10:e002262. doi: 10.1136/bmjsem-2024-002262 39720154 PMC11667426

[37] Spineli LM Kalyvas C Yepes-Nuñez JJ García-Sierra AM Rivera-Pinzón DC Seide SE . Low awareness of the transitivity assumption in complex networks of interventions: a systematic survey from 721 network meta-analyses. BMC Med. (2024) 22:112. doi: 10.1186/s12916-024-03322-1 38475826 PMC10935945

[38] Ades AE Welton NJ Dias S Phillippo DM Caldwell DM . Twenty years of network meta-analysis: continuing controversies and recent developments. Res Synth Methods. (2024) 15:702–27. doi: 10.1002/jrsm.1700 38234221

[39] Pedder H Dias S Bennetts M Boucher M Welton NJ . Modelling time-course relationships with multiple treatments: Model-based network meta-analysis for continuous summary outcomes. Res Synth Methods. (2019) 10:267–86. doi: 10.1002/jrsm.1351 31013000 PMC6563489

[40] Borg DN Impellizzeri FM Borg SJ Hutchins KP Stewart IB Jones T . Meta-analysis prediction intervals are under reported in sport and exercise medicine. Scand J Med Sci Sports. (2024) 34:e14603. doi: 10.1111/sms.14603 38501202

[41] Brignardello-Petersen R Guyatt GH . Introduction to network meta-analysis: understanding what it is, how it is done, and how it can be used for decision-making. Am J Epidemiol. (2025) 194:837–43. doi: 10.1093/aje/kwae260 39108176 PMC11879513

[42] Veroniki AA Tricco AC Rangira D McKenzie JE Li T Straus SE . Updating the PRISMA reporting guideline for network meta-analysis: a scoping review. J Clin Epidemiol. (2025) 188:111985. doi: 10.1016/j.jclinepi.2025.111985 40976519

[43] Zhu JY Guo L . Exercise-regulated lipolysis: its role and mechanism in health and diseases. J Adv Res. (2025) 75:291–309. doi: 10.1016/j.jare.2024.11.031 39613256 PMC12536597

[44] Ashcroft SP Stocks B Egan B Zierath JR . Exercise induces tissue-specific adaptations to enhance cardiometabolic health. Cell Metab. (2024) 36:278–300. doi: 10.1016/j.cmet.2023.12.008 38183980

[45] Hoffmann WG Chen YQ Schwartz CS Barber JL Dev PK Reasons RJ . Effects of exercise training on ANGPTL3/8 and ANGPTL4/8 and their associations with cardiometabolic traits. J Lipid Res. (2024) 65:100495. doi: 10.1016/j.jlr.2023.100495 38160757 PMC10832466

[46] Boutcher SH . High-intensity intermittent exercise and fat loss. J Obes. (2011) 2011:868305. doi: 10.1155/2011/868305 21113312 PMC2991639

[47] Feng J Zhang Q Chen B Chen J Wang W Hu Y . Effects of high-intensity intermittent exercise on glucose and lipid metabolism in type 2 diabetes patients: a systematic review and meta-analysis. Front Endocrinol. (2024) 15:1360998. doi: 10.3389/fendo.2024.1360998 38978627 PMC11229039

[48] Fan J Zhang X Zhang J Zhao T Burley SK Zheng XFS . PDX1 phosphorylation at S61 by mTORC1 links nutrient signaling to β cell function and metabolic disease. Cell Rep. (2026) 45:116811. doi: 10.1016/j.celrep.2025.116811 41528843 PMC12949489

[49] Fan J Yuan Z Burley SK Libutti SK Zheng XFS . Amino acids control blood glucose levels through mTOR signaling. Eur J Cell Biol. (2022) 101:151240. doi: 10.1016/j.ejcb.2022.151240 35623230 PMC10035058

[50] Fan J Xu Y . Molecular mechanisms underlying the lifespan and healthspan benefits of dietary restriction across species. Front Genet. (2026) 17:1771707. doi: 10.3389/fgene.2026.1771707 41695773 PMC12900492

[51] Fan J Gao Y Lu Y Wu W Yuan S Wu H . PKAc-directed interaction and phosphorylation of Ptc is required for Hh signaling inhibition in Drosophila. Cell Discov. (2019) 5:44. doi: 10.1038/s41421-019-0112-z 31636957 PMC6796939

[52] Liu Y Dong G Zhao X Huang Z Li P Zhang H . Post-exercise effects and long-term training adaptations of hormone sensitive lipase lipolysis induced by high-intensity interval training in adipose tissue of mice. Front Physiol. (2020) 11:535722. doi: 10.3389/fphys.2020.535722 33324231 PMC7723847

[53] Magalhaes JP Santos DA Correia IR Hetherington-Rauth M Ribeiro R Raposo JF . Impact of combined training with different exercise intensities on inflammatory and lipid markers in type 2 diabetes: a secondary analysis from a 1-year randomized controlled trial. Cardiovasc Diabetol. (2020) 19:169. doi: 10.1186/s12933-020-01136-y 33028418 PMC7539392

[54] Xing S Zhang Y Chen Y Feng S Zhang Y Moreira P . Comparing the impacts of different exercise interventions on patients with type 2 diabetes mellitus: a literature review and meta-analysis. Front Endocrinol. (2025) 16:1495131. doi: 10.3389/fendo.2025.1495131 40391012 PMC12086073

[55] Duft RG Bonfante ILP Palma-Duran SA Chacon-Mikahil MPT Griffin JL Cavaglieri CR . Moderate-intensity combined training induces lipidomic changes in individuals with obesity and type 2 diabetes. J Clin Endocrinol Metab. (2024) 109:2182–98. doi: 10.1210/clinem/dgae177 38488044 PMC11318996

[56] Nomikos T Methenitis S Panagiotakos DB . The emerging role of skeletal muscle as a modulator of lipid profile the role of exercise and nutrition. Lipids Health Dis. (2022) 21:81. doi: 10.1186/s12944-022-01692-0 36042487 PMC9425975

[57] Ginsberg HN Packard CJ Chapman MJ Borén J Aguilar-Salinas CA Averna M . Triglyceride-rich lipoproteins and their remnants: metabolic insights, role in atherosclerotic cardiovascular disease, and emerging therapeutic strategies-a consensus statement from the European Atherosclerosis Society. Eur Heart J. (2021) 42:4791–806. doi: 10.1093/eurheartj/ehab551 34472586 PMC8670783

[58] Jayaraman S Pérez A Miñambres I Sánchez-Quesada JL Gursky O . Heparin binding triggers human VLDL remodeling by circulating lipoprotein lipase: Relevance to VLDL functionality in health and disease. Biochim Biophys Acta Mol Cell Biol Lipids. (2022) 1867:159064. doi: 10.1016/j.bbalip.2021.159064 34610468 PMC8595799

[59] Ghazvineh D Daneshvar M Basirat V Daneshzad E . The effect of yoga on the lipid profile: a systematic review and meta-analysis of randomized clinical trials. Front Nutr. (2022) 9:942702. doi: 10.3389/fnut.2022.942702 35911119 PMC9329825

[60] Li JX Cummins CL . Fresh insights into glucocorticoid-induced diabetes mellitus and new therapeutic directions. Nat Rev Endocrinol. (2022) 18:540–57. doi: 10.1038/s41574-022-00683-6 35585199 PMC9116713

[61] Viswanathan V Sivakumar S Sai Prathiba A Devarajan A George L Kumpatla S . Effect of yoga intervention on biochemical, oxidative stress markers, inflammatory markers and sleep quality among subjects with type 2 diabetes in South India: Results from the SATYAM project. Diabetes Res Clin Pract. (2021) 172:108644. doi: 10.1016/j.diabres.2020.108644 33359750

[62] Chen S Deng S Liu Y Yin T . Effects of yoga on blood glucose and lipid profile of type 2 diabetes patients without complications: a systematic review and meta-analysis. Front Sports Act Living. (2022) 4:900815. doi: 10.3389/fspor.2022.900815 35813055 PMC9259958

[63] Chattopadhyay K Mishra P Singh K Singh K Harris T Hamer M . Yoga programme for type 2 diabetes prevention (YOGA-DP) among high-risk people in india: a multicenter feasibility randomized controlled trial. Diabetes Ther. (2023) 14:1137–54. doi: 10.1007/s13300-023-01395-4 37002475 PMC10066010

[64] Powers SK Deminice R Ozdemir M Yoshihara T Bomkamp MP Hyatt H . Exercise-induced oxidative stress: Friend or foe? J Sport Health Sci. (2020) 9:415–25. doi: 10.1016/j.jshs.2020.04.001 32380253 PMC7498668

[65] Meng Q Su CH . The impact of physical exercise on oxidative and nitrosative stress: balancing the benefits and risks. Antioxidants (Basel). (2024) 13:573. doi: 10.3390/antiox13050573 PMC11118032 38790678

[66] Chen Y Jin X Chen G Wang R Tian H . Dose-response relationship between physical activity and the morbidity and mortality of cardiovascular disease among individuals with diabetes: meta-analysis of prospective cohort studies. JMIR Public Health Surveill. (2024) 10:e54318. doi: 10.2196/54318 38780218 PMC11369533

[67] Urhausen A Kindermann W . Diagnosis of overtraining. Sports Med. (2002) 32:95–102. doi: 10.2165/00007256-200232020-00002 11817995

[68] Adiels M Olofsson SO Taskinen MR Borén J . Overproduction of very low-density lipoproteins is the hallmark of the dyslipidemia in the metabolic syndrome. Arterioscler Thromb Vasc Biol. (2008) 28:1225–36. doi: 10.1161/atvbaha.107.160192 18565848

[69] Petitt DS Cureton KJ . Effects of prior exercise on postprandial lipemia: a quantitative review. Metabolism. (2003) 52:418–24. doi: 10.1053/meta.2003.50071 12701052

[70] Bays HE Kirkpatrick C Maki KC Toth PP Morgan RT Tondt J . Obesity, dyslipidemia, and cardiovascular disease: A joint expert review from the Obesity Medicine Association and the National Lipid Association 2024. Obes Pillars. (2024) 10:100108. doi: 10.1016/j.obpill.2024.100108 38706496 PMC11066689

[71] Li M Chi X Wang Y Setrerrahmane S Xie W Xu H . Trends in insulin resistance: insights into mechanisms and therapeutic strategy. Signal Transduct Target Ther. (2022) 7:216. doi: 10.1038/s41392-022-01073-0 35794109 PMC9259665

[72] Jensen MD Ryan DH Apovian CM Ard JD Comuzzie AG Donato KA . 2013 AHA/ACC/TOS guideline for the management of overweight and obesity in adults: a report of the American College of Cardiology/American Heart Association Task Force on Practice Guidelines and The Obesity Society. J Am Coll Cardiol. (2014) 63:2985–3023. doi: 10.1016/j.jacc.2013.11.004 24239920

[73] American Diabetes Association Professional Practice Committee . 6. Glycemic goals and hypoglycemia: standards of care in diabetes-2025. Diabetes Care. (2025) 48:S128–S45. doi: 10.2337/dc25-S006 39651981 PMC11635034

[74] American Diabetes Association Professional Practice Committee . 10. Cardiovascular disease and risk management: standards of care in diabetes-2025. Diabetes Care. (2025) 48:S207–S38. doi: 10.2337/dc25-S010 39651970 PMC11635050

