# Supplementary material for: Comparative effects of different exercise modalities and doses on lipid metabolism in patients with type 2 diabetes mellitus: a systematic review with Bayesian dose–response network meta-analysis
Source: Front Endocrinol (Lausanne). 2026 Oct 7;17:1947147. doi: 10.3389/fendo.2026.1947147 (PMC13683165; doi:10.3389/fendo.2026.1947147)
Supplement: Supplementary file 1 [file Supplementaryfile1.docx]

**Supplementary Material**

**Contents**

[1. PRISMA NMA Checklist of Items to Include When Reporting A Systematic Review Involving A Network Meta-analysis	2](#_Toc1115)

[2. Search strategy	6](#_Toc11147)

[3. Demographic characteristics of included studies	9](#_Toc6417)

[4. Risk of bias	24](#_Toc30574)

[5. Confidence of evidence	27](#_Toc14079)

[6. Pairwise meta-analysis and publication bias	28](#_Toc14851)

[6.1 Details of pairwise meta-analysis	28](#_Toc14020)

[6.2 Publication bias	28](#_Toc3653)

[7. Bayesian network meta-analysis	29](#_Toc22115)

[7.1 Model fit summaries	29](#_Toc17358)

[7.2 Node-split method for assessment of inconsistency	30](#_Toc4072)

[7.3 Forest plots showing the effects of exercise modality on blood lipid levels	32](#_Toc21497)

[7.4 SUCRA table for all studies	33](#_Toc7766)

[7.5 Assessment of transitivity assumption	34](#_Toc3202)

[7.6 Sensitivity analysis	36](#_Toc26472)

[7.7 Network meta-regressions	39](#_Toc32483)

[8. Dose-response network meta-analysis	41](#_Toc13978)

[8.1 Key assumptions for network meta-analysis	41](#_Toc28915)

[8.2 Model selection	48](#_Toc18848)

[8.3 Dose-response relationships	56](#_Toc25568)

**1. PRISMA NMA Checklist of Items to Include When Reporting A Systematic Review** **Involving A Network Meta-analysis**

| **Section/Topic** | **Item #** | **Checklist Item** | **Reported on Page #** |
| --- | --- | --- | --- |
| **TITLE** |  |  |  |
| Title | 1 | Identify the report as a systematic review *incorporating a network meta-analysis (or related form of meta-analysis).* | ***1*** |
|  |  |  |  |
| **ABSTRACT** |  |  |  |
| Structured summary | 2 | Provide a structured summary including, as applicable:  **Background:** main objectives  **Methods:** data sources; study eligibility criteria, participants, and interventions; study appraisal; and *synthesis methods, such as network meta-analysis.*  **Results:** number of studies and participants identified; summary estimates with corresponding confidence/credible intervals; *treatment rankings may also be discussed. Authors may choose to summarize pairwise comparisons against a chosen treatment included in their analyses for brevity.*  **Discussion/Conclusions:** limitations; conclusions and implications of findings.  **Other:** primary source of funding; systematic review registration number with registry name. | 1-2 |
|  |  |  |  |
| **INTRODUCTION** |  |  |  |
| Rationale | 3 | Describe the rationale for the review in the context of what is already known*, including mention of why a network meta-analysis has been conducted.* | ***2*** |
| Objectives | 4 | Provide an explicit statement of questions being addressed, with reference to participants, interventions, comparisons, outcomes, and study design (PICOS). | 2 |
|  |  |  |  |
| **METHODS** |  |  |  |
| Protocol and registration | 5 | Indicate whether a review protocol exists and if and where it can be accessed (e.g., Web address); and, if available, provide registration information, including registration number. | 3 |
| Eligibility criteria | 6 | Specify study characteristics (e.g., PICOS, length of follow-up) and report characteristics (e.g., years considered, language, publication status) used as criteria for eligibility, giving rationale. *Clearly describe eligible treatments included in the treatment network, and note whether any have been clustered or merged into the same node (with justification).* | ***3*** |
| Information sources | 7 | Describe all information sources (e.g., databases with dates of coverage, contact with study authors to identify additional studies) in the search and date last searched. | 3 |
| Search | 8 | Present full electronic search strategy for at least one database, including any limits used, such that it could be repeated. | Supplementary Material 2 |
| Study selection | 9 | State the process for selecting studies (i.e., screening, eligibility, included in systematic review, and, if applicable, included in the meta-analysis). | 3 |
| Data collection process | 10 | Describe method of data extraction from reports (e.g., piloted forms, independently, in duplicate) and any processes for obtaining and confirming data from investigators. | 3 |
| Data items | 11 | List and define all variables for which data were sought (e.g., PICOS, funding sources) and any assumptions and simplifications made. | Supplementary Material 3 |
| **Geometry of the network** | **S1** | Describe methods used to explore the geometry of the treatment network under study and potential biases related to it. This should include how the evidence base has been graphically summarized for presentation, and what characteristics were compiled and used to describe the evidence base to readers. | ***4-6*** |
| Risk of bias within individual studies | 12 | Describe methods used for assessing risk of bias of individual studies (including specification of whether this was done at the study or outcome level), and how this information is to be used in any data synthesis. | 3 |
| Summary measures | 13 | State the principal summary measures (e.g., risk ratio, difference in means). *Also describe the use of additional summary measures assessed, such as treatment rankings and surface under the cumulative ranking curve (SUCRA) values, as well as modified approaches used to present summary findings from meta-analyses.* | 4 |
| Planned methods of analysis | 14 | Describe the methods of handling data and combining results of studies for each network meta-analysis. This should include, but not be limited to:   - *Handling of multi-arm trials;* - *Selection of variance structure;* - *Selection of prior distributions in Bayesian analyses; and* - *Assessment of model fit.* | 4 |
| **Assessment of Inconsistency** | **S2** | Describe the statistical methods used to evaluate the agreement of direct and indirect evidence in the treatment network(s) studied. Describe efforts taken to address its presence when found. | 4; Supplementary Material 7.1–7.2 |
| Risk of bias across studies | 15 | Specify any assessment of risk of bias that may affect the cumulative evidence (e.g., publication bias, selective reporting within studies). | **4; Supplementary Material 6.2** |
| Additional analyses | 16 | Describe methods of additional analyses if done, indicating which were pre-specified. This may include, but not be limited to, the following:   - Sensitivity or subgroup analyses; - Meta-regression analyses; - *Alternative formulations of the treatment network; and* - *Use of alternative prior distributions for Bayesian analyses (if applicable).* | ***4*** |
|  |  |  |  |
| **RESULTS†** |  |  |  |
| Study selection | 17 | Give numbers of studies screened, assessed for eligibility, and included in the review, with reasons for exclusions at each stage, ideally with a flow diagram. | 4-5 |
| **Presentation of network structure** | **S3** | Provide a network graph of the included studies to enable visualization of the geometry of the treatment network. | ***6*** |
| **Summary of network geometry** | **S4** | Provide a brief overview of characteristics of the treatment network. This may include commentary on the abundance of trials and randomized patients for the different interventions and pairwise comparisons in the network, gaps of evidence in the treatment network, and potential biases reflected by the network structure. | ***5-6*** |
| Study characteristics | 18 | For each study, present characteristics for which data were extracted (e.g., study size, PICOS, follow-up period) and provide the citations. | 4; Supplementary Material 3 |
| Risk of bias within studies | 19 | Present data on risk of bias of each study and, if available, any outcome level assessment. | 5; Supplementary Material 4 |
| Results of individual studies | 20 | For all outcomes considered (benefits or harms), present, for each study: 1) simple summary data for each intervention group, and 2) effect estimates and confidence intervals. *Modified approaches may be needed to deal with information from larger networks.* | ***5-7; Table 1 on p. 7; Supplementary Material 6-7*** |
| Synthesis of results | 21 | Present results of each meta-analysis done, including confidence/credible intervals. *In larger networks, authors may focus on comparisons versus a particular comparator (e.g. placebo or standard care), with full findings presented in an appendix. League tables and forest plots may be considered to summarize pairwise comparisons.* If additional summary measures were explored (such as treatment rankings), these should also be presented. | ***5-8; Supplementary Material 6-8*** |
| **Exploration for inconsistency** | **S5** | Describe results from investigations of inconsistency. This may include such information as measures of model fit to compare consistency and inconsistency models, *P* values from statistical tests, or summary of inconsistency estimates from different parts of the treatment network. | ***Supplementary Material 7.1-7.2*** |
| Risk of bias across studies | 22 | Present results of any assessment of risk of bias across studies for the evidence base being studied. | 5; Supplementary Material 6.2 |
| Results of additional analyses | 23 | Give results of additional analyses, if done (e.g., sensitivity or subgroup analyses, meta-regression analyses*, alternative network geometries studied, alternative choice of prior distributions for Bayesian analyses,* and so forth). | ***6-7; Supplementary Material 7.6–7.7*** |
|  |  |  |  |
| **DISCUSSION** |  |  |  |
| Summary of evidence | 24 | Summarize the main findings, including the strength of evidence for each main outcome; consider their relevance to key groups (e.g., healthcare providers, users, and policy-makers). | 7-11 |
| Limitations | 25 | Discuss limitations at study and outcome level (e.g., risk of bias), and at review level (e.g., incomplete retrieval of identified research, reporting bias). *Comment on the validity of the assumptions, such as transitivity and consistency. Comment on any concerns regarding network geometry (e.g., avoidance of certain comparisons).* | 11 |
| Conclusions | 26 | Provide a general interpretation of the results in the context of other evidence, and implications for future research. | 11 |
|  |  |  |  |
| **FUNDING** |  |  |  |
| Funding | 27 | Describe sources of funding for the systematic review and other support (e.g., supply of data); role of funders for the systematic review. This should also include information regarding whether funding has been received from manufacturers of treatments in the network and/or whether some of the authors are content experts with professional conflicts of interest that could affect use of treatments in the network. | ***11*** |

PICOS = population, intervention, comparators, outcomes, study design.

* Text in italics indicates wording specific to reporting of network meta-analyses that has been added to guidance from the PRISMA statement.

† Authors may wish to plan for use of appendices to present all relevant information in full detail for items in this section.

**2. Search strategy**

**PubMed**

#1 "Diabetes Mellitus, Type 2"[Mesh]

#2 type 2 diabet*[tiab] OR T2DM[tiab] OR NIDDM[tiab] OR non-insulin dependent diabet*[tiab] OR noninsulin dependent diabet*[tiab] OR adult-onset diabet*[tiab] OR maturity-onset diabet*[tiab]

#3 #1 OR #2

#4 "Exercise"[Mesh] OR "Exercise Therapy"[Mesh] OR "Resistance Training"[Mesh] OR "High-Intensity Interval Training"[Mesh] OR "Walking"[Mesh] OR "Running"[Mesh]

#5 exercise*[tiab] OR physical activ*[tiab] OR exercise training[tiab] OR aerobic exercise[tiab] OR aerobic training[tiab] OR endurance training[tiab] OR walking[tiab] OR running[tiab] OR treadmill[tiab] OR cycling[tiab]

#6 resistance training[tiab] OR strength training[tiab] OR weight training[tiab] OR weight lifting[tiab]

#7 combined training[tiab] OR concurrent training[tiab] OR combined exercise[tiab] OR multicomponent exercise[tiab] OR aerobic and resistance[tiab] OR aerobic plus resistance[tiab] OR aerobic combined resistance[tiab]

#8 high-intensity interval training[tiab] OR high intensity interval training[tiab] OR HIIT[tiab] OR interval training[tiab] OR sprint interval training[tiab]

#9 yoga[tiab] OR pilates[tiab]

#10 tai chi[tiab] OR tai-chi[tiab] OR taiji[tiab] OR taijiquan[tiab] OR qigong[tiab] OR qi gong[tiab] OR baduanjin[tiab] OR ba duan jin[tiab] OR traditional Chinese exercise[tiab] OR Chinese traditional exercise[tiab] OR Chinese exercise[tiab]

#11 dance[tiab] OR dancing[tiab]

#12 #4 OR #5 OR #6 OR #7 OR #8 OR #9 OR #10 OR #11

#13 randomized controlled trial[pt] OR controlled clinical trial[pt]

#14 random*[tiab] OR trial[tiab] OR clinical trial[tiab] OR RCT[tiab] OR crossover[tiab] OR cross-over[tiab]

#15 #13 OR #14

#16 #3 AND #12 AND #15

**Embase**

#1 'type 2 diabetes mellitus'/exp

#2 'type 2 diabet*':ti,ab OR t2dm:ti,ab OR niddm:ti,ab OR 'non insulin dependent diabet*':ti,ab OR 'non-insulin dependent diabet*':ti,ab OR 'adult onset diabet*':ti,ab OR 'adult-onset diabet*':ti,ab OR 'maturity onset diabet*':ti,ab OR 'maturity-onset diabet*':ti,ab

#3 #1 OR #2

#4 'exercise'/exp OR 'exercise therapy'/exp OR 'resistance training'/exp OR 'high intensity interval training'/exp OR 'walking'/exp OR 'running'/exp

#5 exercise*:ti,ab OR 'physical activ*':ti,ab OR 'exercise training':ti,ab OR 'aerobic exercise':ti,ab OR 'aerobic training':ti,ab OR 'endurance training':ti,ab OR walking:ti,ab OR running:ti,ab OR treadmill:ti,ab OR cycling:ti,ab

#6 'resistance training':ti,ab OR 'strength training':ti,ab OR 'weight training':ti,ab OR 'weight lifting':ti,ab

#7 'combined training':ti,ab OR 'concurrent training':ti,ab OR 'combined exercise':ti,ab OR 'multicomponent exercise':ti,ab OR 'aerobic and resistance':ti,ab OR 'aerobic plus resistance':ti,ab OR 'aerobic combined resistance':ti,ab

#8 'high-intensity interval training':ti,ab OR 'high intensity interval training':ti,ab OR hiit:ti,ab OR 'interval training':ti,ab OR 'sprint interval training':ti,ab

#9 yoga:ti,ab OR pilates:ti,ab

#10 'tai chi':ti,ab OR 'tai-chi':ti,ab OR taiji:ti,ab OR taijiquan:ti,ab OR qigong:ti,ab OR 'qi gong':ti,ab OR baduanjin:ti,ab OR 'ba duan jin':ti,ab OR 'traditional Chinese exercise':ti,ab OR 'Chinese traditional exercise':ti,ab OR 'Chinese exercise':ti,ab

#11 dance:ti,ab OR dancing:ti,ab

#12 #4 OR #5 OR #6 OR #7 OR #8 OR #9 OR #10 OR #11

#13 'randomized controlled trial'/exp OR 'controlled clinical trial'/exp OR 'clinical trial'/exp

#14 random*:ti,ab OR trial*:ti,ab OR rct:ti,ab OR crossover:ti,ab OR 'cross-over':ti,ab

#15 #13 OR #14

#16 #3 AND #12 AND #15

**Cochrane Library**

#1 MeSH descriptor: [Diabetes Mellitus, Type 2] explode all trees

#2 (type 2 diabet* OR T2DM OR NIDDM OR non-insulin dependent diabet* OR noninsulin dependent diabet* OR adult-onset diabet* OR maturity-onset diabet*):ti,ab,kw

#3 #1 OR #2

#4 MeSH descriptor: [Exercise] explode all trees OR MeSH descriptor: [Exercise Therapy] explode all trees OR MeSH descriptor: [Resistance Training] explode all trees OR MeSH descriptor: [High-Intensity Interval Training] explode all trees OR MeSH descriptor: [Walking] explode all trees OR MeSH descriptor: [Running] explode all trees

#5 (exercise* OR physical activ* OR exercise training OR aerobic exercise OR aerobic training OR endurance training OR walking OR running OR treadmill OR cycling):ti,ab,kw

#6 (resistance training OR strength training OR weight training OR weight lifting):ti,ab,kw

#7 (combined training OR concurrent training OR combined exercise OR multicomponent exercise OR aerobic and resistance OR aerobic plus resistance OR aerobic combined resistance):ti,ab,kw

#8 (high-intensity interval training OR high intensity interval training OR HIIT OR interval training OR sprint interval training):ti,ab,kw

#9 (yoga OR pilates):ti,ab,kw

#10 (tai chi OR tai-chi OR taiji OR taijiquan OR qigong OR qi gong OR baduanjin OR ba duan jin OR traditional Chinese exercise OR Chinese traditional exercise OR Chinese exercise):ti,ab,kw

#11 (dance OR dancing):ti,ab,kw

#12 #4 OR #5 OR #6 OR #7 OR #8 OR #9 OR #10 OR #11

#13 random* OR trial* OR RCT OR crossover OR cross-over

#14 #3 AND #12

#15 #14 AND #13

**Web of Science**

#1 TS=("type 2 diabet*" OR T2DM OR NIDDM OR "non-insulin dependent diabet*" OR "noninsulin dependent diabet*" OR "adult-onset diabet*" OR "adult onset diabet*" OR "maturity-onset diabet*" OR "maturity onset diabet*")

#2 TS=(exercise* OR "physical activ*" OR "exercise training" OR "aerobic exercise" OR "aerobic training" OR "endurance training" OR walking OR running OR treadmill OR cycling)

#3 TS=("resistance training" OR "strength training" OR "weight training" OR "weight lifting")

#4 TS=("combined training" OR "concurrent training" OR "combined exercise" OR "multicomponent exercise" OR "aerobic and resistance" OR "aerobic plus resistance" OR "aerobic combined resistance")

#5 TS=("high-intensity interval training" OR "high intensity interval training" OR HIIT OR "interval training" OR "sprint interval training")

#6 TS=(yoga OR pilates)

#7 TS=("tai chi" OR "tai-chi" OR taiji OR taijiquan OR qigong OR "qi gong" OR baduanjin OR "ba duan jin" OR "traditional Chinese exercise" OR "Chinese traditional exercise" OR "Chinese exercise")

#8 TS=(dance OR dancing)

#9 #2 OR #3 OR #4 OR #5 OR #6 OR #7 OR #8

#10 TS=(random* OR trial* OR RCT OR crossover OR "cross-over")

#11 #1 AND #9 AND #10

3. **Demographic characteristics of included studies**

| **Author/**  **Year** | **Intervention** | **Country** | **Outcomes** | **Sample size** | **Sex (male/female)** | **Age (x±s)** | **BMI (x±s)** | **Duration of diabetes (years)** | **Duration (weeks)** | **Frequency (sessions/week)** | **Session duration (min)** | **Medication changes** | **Diet changes** | **Insulin  treatment** | **Adherence reporting** | **Analysis population** |
| --- | --- | --- | --- | --- | --- | --- | --- | --- | --- | --- | --- | --- | --- | --- | --- | --- |
| Wu Meiqiao 2020 | HIIT | China | ①, ② | 32 | 18/14 | 35.06±3.44 | 28.89±0.53 | 16.47±4.57 | 24 | 5 | 20 | Yes | Yes | NR | NR | NR |
|  | CAE |  |  | 32 | 20/12 | 34.56±3.74 | 28.98±0.68 | 17.03±4.03 | 24 | 5 | 30 | Yes | Yes | NR | NR | NR |
| Arora 2007 | CAE | India | ① | 10 | 6/4 | 52.2±9.3 | 26.23±3.2 | 4.7±1.7 | 8 | 3 | 30 | No | No | NR | NR | Completer |
|  | RT |  |  | 9 |  | 49.6±5.2 | 26.99±4.1 | 5.4±1.5 | 8 | 2 | 60 | No | No | NR | NR | Completer |
|  | CG |  |  | 10 | 6/4 | 58.4±1.8 | 24.98±3 | 5.2±2.9 |  |  |  | No | No | NR | Not applicable | Completer |
| Sigal 2007 | CE | Canada | ①, ② | 64 | 40/24 | 53.5±7.3 |  | 5.2±4.8 | 24 | 3 | 55 | Yes | Yes | NR | Reported: Median exercise attendance was approximately 86% in the CE group. Participants were also required to attend at least 10 of 12 run-in sessions before randomization. | ITT |
|  | CAE |  |  | 60 | 39/21 | 53.9±6.6 |  | 5.1±3.5 | 24 | 3 | 35 | Yes | Yes | NR | Reported; Median exercise attendance was approximately 80% in the CAE group. Participants were also required to attend at least 10 of 12 run-in sessions before randomization. | ITT |
|  | RT |  |  | 64 | 40/24 | 54.7±7.5 |  | 6.1±4.7 | 24 | 3 | 20 | Yes | Yes | NR | Reported: Median exercise attendance was approximately 85% in the RT group. Participants were also required to attend at least 10 of 12 run-in sessions before randomization. | ITT |
|  | CG |  |  | 63 | 41/22 | 54.8±7.2 |  | 5.0±4.5 |  |  |  | Yes | Yes | NR | Not applicable | ITT |
| Honkola 1997 | RT | Finland | ①, ② | 18 | 12/6 | 62.0±8.49 |  | 8.0±8.49 | 20 | 2 | 30 | No | No | No | NR | Completer |
|  | CG |  |  | 20 | 5/15 | 67.0±8.94 |  | 8.0±8.94 |  |  |  | No | No | No | Not applicable | Completer |
| Cauza 2006 | CAE | Austria | ①, ② | 10 | 5/5 | 57.1±5.06 |  | 9.1±7.91 | 16 | 3 | 30 | No | No | No | NR | Completer |
|  | RT |  |  | 10 | 5/5 | 56.9±5.06 |  | 9.7±5.69 | 16 | 3 | 20 | No | No | No | NR | Completer |
| Keun Hee An 2005 | RT | USA | ①, ② | 10 |  | 59.2±2.7 | 28.7±1.3 | 7.8±1.7 | 12 | 5 | 50 | NR | NR | NR | NR | NR |
|  | CAE |  |  | 9 |  | 53.1±2.5 | 29.5±1.3 | 6.6±1.4 | 12 | 5 | 50 | NR | NR | NR | NR | NR |
|  | CG |  |  | 7 |  | 52.3±3.3 | 29.2±1.1 | 5.3±1.6 |  |  |  | NR | NR | NR | Not applicable | NR |
| Cauza 2005 | CAE | Austria | ② | 17 | 9/8 | 57.9±5.77 | 33.9±5.36 |  | 16 | 3 | 25 | NR | NR | No | NR | NR |
|  | RT |  |  | 22 | 11/11 | 56.4±5.16 | 31.3±4.22 |  | 16 | 3 | 30 | NR | NR | No | NR | NR |
| Kadoglou 2012 | RT | Greece | ①, ② | 23 | 7/16 | 61.5±5.4 | 32.74±4.05 | 6±2.8 | 12 | 3 | 50 | NR | NR | No | NR | NR |
|  | CG |  |  | 24 | 5/19 | 64.6±4.3 | 31.58±5.71 | 5.6±1.9 |  |  |  | NR | NR | No | Not applicable | NR |
| Kadoglou NP 2012 | CAE | Greece | ①, ② | 26 | 9/17 | 58.4±5.7 | 32.1±3.77 | 5.1±2.5 | 12 | 4 | 60 | NR | NR | No | NR | NR |
|  | CG |  |  | 27 | 10/17 | 62.9±4.2 | 30.04±2.95 | 5.5±2.1 |  |  |  | NR | NR | No | Not applicable | NR |
| Pandey 2017 | HIIT | Canada | ①, ② | 21 | 16/5 | 68±9 | 32.3±2.1 |  | 12 | 5 | 30 | NR | NR | NR | Reported: Exercise adherence was recorded using logs, and self-reported exercise minutes per month were reported. | NR |
|  | CAE |  |  | 19 | 12/7 | 65±9 | 32.4±1.9 |  | 12 | 5 | 30 | NR | NR | NR | Reported: Exercise adherence was recorded using logs, and self-reported exercise minutes per month were reported. | NR |
| Koh 2018 | CAE | Denmark | ①, ② | 8 | 4/4 | 58±9 | 28.6±2.5 | 6±4 | 11 | 3 | 40 | No | No | No | Reported: Training adherence was 98 ± 8% in the CAE group. | Completer |
|  | HIIT |  |  | 8 | 5/3 | 56±5 | 27.6±2.7 | 9±4 | 11 | 3 | 20 | No | No | No | Reported: Training adherence was 97 ± 13% in the HIIT group. | Completer |
| Dede 2015 | CAE | Türkiye | ①, ② | 30 | 15/15 | 52.5±7.5 | 30.8±4.6 | 6.1±5.2 | 12 | 3 | 45 | No | Yes | No | Partially reported: Attendance was monitored by trainers, and participants who trained for fewer than 10 weeks were excluded. Six participants withdrew because of non-adherence or loss to follow-up, but an attendance percentage was not reported. | Completer |
|  | CG |  |  | 30 | 14/16 | 55.5±8.4 | 30.2±4.5 | 6.7±4.5 |  |  |  | No | Yes | No | Not applicable | Completer |
| Rahbar 2017 | CAE | Iran | ①, ② | 13 |  | 48.31±5.02 | 27.4±3.65 | 4.61±2.14 | 8 | 3 | 30 | No | No | NR | Partially reported: Two participants in the exercise group completed 12 sessions but could not continue and were excluded. The final exercise sample was 13 participants, but an attendance percentage was not reported. | PP |
|  | CG |  |  | 15 |  | 48.6±4.8 | 26.93±2.42 | 5.33±1.99 |  |  |  | No | No | NR | Not applicable | PP |
| Liu Shuxiu 2017 | CAE | China | ①, ② | 30 | 0/30 | 58.1±3.6 | 25.3±3.5 | 7.8±1.9 | 24 | 3 | 45 | NR | NR | NR | Partially reported: Participants were described as having good adherence, but session attendance or an adherence percentage was not reported. | Completer |
|  | CG |  |  | 30 | 0/30 | 57.4±4.1 | 24.1±4.2 | 6.9±2.2 |  |  |  | NR | NR | NR | Not applicable | Completer |
| Choi 2012 | CAE | South Korea | ①, ② | 38 | 0/38 | 53.8±7.2 |  |  | 12 | 5 | 60 | No | No | No | NR | Completer |
|  | CG |  |  | 37 | 0/37 | 55±6 |  |  |  |  |  | No | No | No | Not applicable | Completer |
| Tan Sijie 2012 | CE | China | ①, ② | 15 |  | 65.9 ±4.2 | 25.2±2.5 | 16.7±6.7 | 24 | 3 | 50 | No | No | No | NR | Completer |
|  | CG |  |  | 10 |  | 64.8 ±6.8 | 25.8±2.5 | 15.4±7.3 |  |  |  | No | No | No | Not applicable | Completer |
| Nagarathna 2012 | Yoga | India | ①, ② | 141 | 91/50 | 53.46±8.86 |  | 6.19±5.49 | 12 | 5 | 60 | Yes | Yes | NR | NR | ITT |
|  | CAE |  |  | 136 | 99/37 | 51.38±8.39 |  | 4.75±4.18 | 12 | 5 | 60 | Yes | Yes | NR | NR | ITT |
| Yang Dan 2020 | CAE | China | ①, ② | 30 | 16/14 | 47.73±7.36 | 26.64±2.94 |  | 12 | 4 | 40 | Yes | Yes | No | Partially reported: Training was recorded using exercise logs, but an attendance percentage or adherence rate was not reported. | Completer |
|  | CG |  |  | 20 | 10/10 | 45.20±8.82 | 25.25±2.18 |  |  |  |  | Yes | Yes | No | Not applicable | Completer |
| Kim 2006 | CAE | South Korea | ①, ② | 32 | 6/26 | 55.0±8.1 | 25.8±3.8 | 7.9±6.5 | 24 | 3 | 75 | Yes | Yes | NR | NR | NR |
|  | CG |  |  | 26 | 8/18 | 53.8±9.0 | 26.2±4.0 | 10.0±6.6 |  |  |  | Yes | Yes | NR | Not applicable | NR |
| Li Songqian 2021 | TCS | China | ①, ② | 10 | 5/5 | 46.2±7.5 | 22.16±3.24 | 5.15±3.04 | 14 | 7 | 60 | No | NR | NR | NR | NR |
|  | CG |  |  | 10 | 4/6 | 44.9±7.49 | 23.59±2.4 | 6.4±4.69 |  |  |  | No | NR | NR | Not applicable | NR |
| Li Kelei 2020 | CAE | China | ①, ② | 20 | 20/0 | 54.17±3.59 | 26.16±1.61 |  | 24 | 3 | 60 | No | NR | No | NR | Completer |
|  | TCS |  |  | 20 | 20/0 | 53.93±3.66 | 26.07±1.83 |  | 24 | 3 | 60 | No | NR | No | NR | Completer |
|  | CG |  |  | 20 | 20/0 | 53.48±3.82 | 25.93±1.77 |  |  |  |  | No | NR | No | Not applicable | Completer |
| Zhao Gang 2017 | TCS | China | ①, ② | 8 |  | 54.75±6.09 | 28.61±1.94 |  | 16 | 7 | 60 | NR | NR | No | NR | Completer |
|  | CG |  |  | 8 |  | 52.38±7.65 | 29.04±2.6 |  |  |  |  | NR | NR | No | Not applicable | Completer |
| Meng En 2014 | TCS | China | ①, ② | 100 | 50/50 | 68.4±3.2 | 26.7±1.5 | 2-23 | 12 | 7 | 45 | NR | Yes | NR | NR | NR |
|  | CG |  |  | 100 | 50/50 | 68.4±3.2 | 26.7±1.5 | 2-23 |  |  |  | NR | Yes | NR | Not applicable | NR |
| Jiang Rongkang 2007 | TCS | China | ①, ② | 18 | 6/12 | 57.2±4.2 | 30.5±5.8 | 3.2±0.8 | 20 | 7 | 45 | NR | NR | NR | NR | NR |
|  | CG |  |  | 18 | 7/11 | 55±6.2 | 28.9±6.2 | 2.9±0.7 |  |  |  | NR | NR | NR | Not applicable | NR |
| Zhu Yunfei 2023 | TCS | China | ①, ② | 10 | 4/6 | 53.8±2.44 |  | 4.66±1.35 | 14 | 6 | 60 | NR | NR | NR | NR | NR |
|  | CG |  |  | 10 | 7/3 | 55.7±2.21 |  | 4.54±1.74 |  |  |  | NR | NR | NR | Not applicable | NR |
| Li Xiaoxiao 2023 | TCS | China | ①, ② | 15 | 9/6 | 55.6±7.5 |  | 2.76±1.21 | 12 | 5 | 60 | NR | Yes | NR | NR | NR |
|  | CG |  |  | 15 | 10/5 | 59.3±7.6 |  | 3.15±1.37 |  |  |  | NR | Yes | NR | Not applicable | NR |
| Qi Yonghao 2023 | TCS | China | ①, ② | 15 | 9/6 | 52.4±7.35 | 21.5±2.87 |  | 12 | 5 | 60 | No | NR | NR | NR | NR |
|  | CG |  |  | 15 | 8/7 | 53.5±5.63 | 20.53±5.17 |  |  |  |  | No | NR | NR | Not applicable | NR |
| Zhao Tianqi 2022 | TCS | China | ①, ② | 12 |  | 67.43±1.77 | 25.67±0.97 | 8.48±2.47 | 12 | 7 | 60 | Yes | NR | NR | NR | NR |
|  | CG |  |  | 12 |  | 67.78±1.93 | 25.29±1.23 | 8.22±2.56 |  |  |  | Yes | NR | NR | Not applicable | NR |
| He Ke 2019 | TCS | China | ①, ② | 24 | 12/12 | 51.83±14.32 | 25.58±3.87 | 5.69±1.94 | 48 | 3 | 45 | NR | NR | NR | NR | NR |
|  | CE |  |  | 27 | 14/13 | 49.36±11.54 | 27.12±2.85 | 4.48±1.11 | 48 | 3 | 65 | NR | NR | NR | NR | NR |
|  | CG |  |  | 24 | 13/11 | 51.59±11.46 | 25.70±2.20 | 6.88±1.08 |  |  |  | NR | NR | NR | Not applicable | NR |
| Guo Qianqian 2019 | TCS | China | ①, ② | 13 | 5/8 | 57.77±6.89 | 25.40±2.74 | 10.27±7.63 | 12 | 4 | 40 | No | No | NR | NR | Completer |
|  | CG |  |  | 15 | 5/10 | 57.53±6.14 | 25.44±4.07 | 11.60±6.24 |  |  |  | No | No | NR | Not applicable | Completer |
| Li Yue 2017 | TCS | China | ①, ② | 43 | 23/20 | 44.32±5.07 |  | 2.13±0.47 | 12 | 4 | 30 | Yes | NR | NR | NR | NR |
|  | CG |  |  | 43 | 22/21 | 42.91±6.10 |  | 2.15±0.52 |  |  |  | Yes | NR | NR | Not applicable | NR |
| Wang Chengyuan 2015 | TCS | China | ①, ② | 30 | 16/14 | 61.7±6.9 |  | 10.6±4.1 | 6 | 5 | 20 | Yes | NR | NR | NR | NR |
|  | CG |  |  | 30 | 17/13 | 61.3±8.4 |  | 10.2±3.6 |  |  |  | Yes | NR | NR | Not applicable | NR |
| Xu Yuxin 2019 | CAE | China | ①, ② | 40 | 21/19 | 65.41±5.01 | 24.86±3.89 | 10-22 | 24 | 5 | 30 | NR | NR | NR | NR | NR |
|  | CE |  |  | 44 | 23/21 | 65.46±4.98 | 25.01±4.02 | 10-22 | 24 | 5 | 45 | NR | NR | NR | NR | NR |
|  | CG |  |  | 36 | 18/18 | 66.32±5.35 | 25.66±3.35 | 10-22 |  |  |  | NR | NR | NR | Not applicable | NR |
| Kan Wei 2021 | CAE | China | ① | 200 | 105/95 | 72.33±10.24 | 26.39 ±2.33 | 3-15 | 24 | 14 | 40 | Yes | NR | NR | NR | NR |
|  | CG |  |  | 200 | 105/95 | 76.22±12.42 | 26.49±2.35 | 3-15 |  |  |  | Yes | NR | NR | Not applicable | NR |
| Lu Fulin 2018 | CAE | China | ①, ② | 48 | 0/48 | 57.2±10.1 | 26.34±3.54 | 5.7±3.5 | 48 | 5 | 45 | NR | NR | NR | NR | NR |
|  | CG |  |  | 48 | 0/48 | 57.9±9.6 | 26.26±4.49 | 5.9±2.8 |  |  |  | NR | NR | NR | Not applicable | NR |
| Xu Lianwu 2013 | CAE | China | ① | 58 | 31/27 | 64.6 | 26.38±2.27 | 12.5 | 24 | 7 | 50 | NR | NR | NR | NR | NR |
|  | CG |  |  | 58 | 33/25 | 63.7 | 26.16±2.33 | 11.8 |  |  |  | NR | NR | NR | Not applicable | NR |
| Wang Ping 2019 | TCS | China | ①, ② | 28 | 16/12 | 49.21±11.12 |  | 8.67±7.37 | 24 | 6 | 40 | NR | NR | NR | NR | NR |
|  | CAE |  |  | 26 | 14/12 | 48.83±10.54 |  | 8.32±7.68 | 24 | 6 | 40 | NR | NR | NR | NR | NR |
| Farias 2015 | RT | Chile | ①, ② | 15 |  | 48.1±1.7 | 26.0±2.1 |  | 6 | 3 | 50 | Yes | NR | NR | NR | NR |
|  | CAE |  |  | 15 |  | 48.1±1.7 | 26.0±2.1 |  | 6 | 3 | 50 | Yes | NR | NR | NR | NR |
| Gordon 2008 | Yoga | Jamaica | ①, ② | 77 | 62/15 | 64 |  |  | 24 | 4 | 60 | NR | NR | NR | NR | NR |
|  | CAE |  |  | 77 | 62/15 | 63.9 |  |  | 24 | 4 | 60 | NR | NR | NR | NR | NR |
|  | CG |  |  | 77 | 62/15 | 63.6 |  |  |  |  |  | NR | NR | NR | Not applicable | NR |
| Singh 2008 | Yoga | India | ①, ② | 30 |  | 45±12.5 | 26.12±1.54 | 1-10 | 6 | 5 | 45 | Yes | NR | NR | NR | NR |
|  | CG |  |  | 30 |  | 45±12.5 | 25.83±1.77 | 1-10 |  |  |  | Yes | NR | NR | Not applicable | NR |
| Huang Rongchun 2011 | TCS | China | ①, ② | 30 | 18/12 | 57.8±7.5 |  | 3-10 | 24 | 4 | 60 | Yes | NR | NR | NR | Completer |
|  | CG |  |  | 30 | 20/10 | 56.5 ± 6.9 |  | 3-10 |  |  |  | Yes | NR | NR | Not applicable | Completer |
| Zhang Jie 2015 | CAE | China | ①, ② | 31 | 14/17 | 51.45±11.06 | 26.44±3.03 |  | 12 | 5 | 55 | NR | NR | NR | NR | NR |
|  | CG |  |  | 31 | 15/16 | 50.76±11.89 | 26.92±3.33 |  |  |  |  | NR | NR | NR | Not applicable | NR |
| Chang Feng 2018 | CAE | China | ①, ② | 18 | 8/10 | 53.3±3.8 | 23.8±4.9 | 3.3±2.9 | 24 | 3 | 30 | NR | NR | NR | NR | NR |
|  | CE |  |  | 18 | 9/9 | 53.2±4.0 | 24.7±4.4 | 3.3±2.7 | 24 | 3 | 60 | NR | NR | NR | NR | NR |
|  | CG |  |  | 20 | 10/10 | 54.3±3.9 | 24±3.2 | 3.4±2.9 |  |  |  | NR | NR | NR | Not applicable | NR |
| Hordern 2008 | CE | Australia | ①, ② | 68 | 36/32 | 57±11 | 32.3±5.4 | 5±5 | 4 | 2 | 60 | NR | NR | NR | NR | NR |
|  | CG |  |  | 64 | 34/30 | 65±8 | 30.9±5.8 | 4±5 |  |  |  | NR | NR | NR | Not applicable | NR |
| Praet 2008 | CAE | Netherlands | ①, ② | 49 | 27/22 | 61±9 | 32.1±5.2 | 5±4 | 48 | 3 | 60 | Yes | NR | NR | Partially reported: After 12 months, 18 participants in the CAE group were still actively participating, but session attendance was not reported. | ITT |
|  | CE |  |  | 43 | 20/23 | 59±9 | 32.5±6 | 5±5 | 48 | 3 | 30 | Yes | NR | NR | Partially reported: After 12 months, 19 participants in the CE group were still actively participating, but session attendance was not reported. | ITT |
| Farzanegi 2017 | CAE | Iran | ①, ② | 14 | 0/14 | 50.83±6.79 | 30.71±3.44 |  | 8 | 3 | 60 | NR | NR | No | NR | NR |
|  | RT |  |  | 14 | 0/14 | 53.28±1.17 | 29.76±3.84 |  | 8 | 3 | 60 | NR | NR | No | NR | NR |
|  | CG |  |  | 14 | 0/14 | 50.17±5.34 | 29.37±4.55 |  |  |  |  | NR | NR | No | Not applicable | NR |
| Barone Gibbs 2012 | CE | USA | ①, ② | 49 | 32/17 | 58±5 | 32.3±5.3 |  | 24 | 3 | 65 | NR | NR | NR | NR | NR |
|  | CG |  |  | 63 | 37/26 | 56±6 | 33.5±4.3 |  |  |  |  | NR | NR | NR | Not applicable | NR |
| Kazemi 2024 | HIIT | Iran | ①, ② | 16 | 16/0 | 57.2±2.5 | 27.6±2.2 | 5.2±0.2 | 12 | 3 | 30 | NR | NR | NR | NR | NR |
|  | CG |  |  | 17 | 17/0 | 58.4±3.3 | 28.2±1.6 | 5.5±0.5 |  |  |  | NR | NR | NR | Not applicable | NR |
| Zadeh 2022 | HIIT | Iran | ①, ② | 17 | 17/0 | 52.2±4.8 | 31.8±1.3 |  | 12 | 4 | 25 | NR | NR | NR | NR | NR |
|  | RT |  |  | 18 | 18/0 | 51.6±5 | 31.6±1.6 |  | 12 | 4 | 30 | NR | NR | NR | NR | NR |
|  | CAE |  |  | 17 | 17/0 | 52.8±3.1 | 31.2±1.1 |  | 12 | 4 | 20 | NR | NR | NR | NR | NR |
|  | CE |  |  | 16 | 16/0 | 53.2±3.9 | 32.2±2 |  | 12 | 4 | 50 | NR | NR | NR | NR | NR |
| Al Ozairi 2023 | RT | Kuwait | ①, ② | 64 | 41/23 | 59.45±8.55 | 30.56±5.15 |  | 32 | 3 | 30 | Yes | NR | No | Partially reported: Adherence was recorded using online exercise logs, but a clear attendance percentage or adherence rate was not reported. | modified ITT + PP |
|  | CG |  |  | 56 | 33/23 | 60.99±10.32 | 31.84±5.7 |  |  |  |  | Yes | NR | No | Not applicable | modified ITT + PP |
| Ma Xiaojun 2024 | CAE | China | ①, ② | 30 | 16/14 | 57.73±5.85 | 26.56±1.55 | ＞2 | 24 | 3 | 60 | No | Yes | NR | Reported: Attendance was 82.22 ± 2.47% in the CAE group. Participants were required to attend more than 70% of sessions, and missed sessions could be made up. | Completer |
|  | RT |  |  | 32 | 16/16 | 57.56±4.85 | 26.33±1.76 | ＞2 | 24 | 3 | 40 | No | Yes | NR | Reported: Attendance was 81.96 ± 2.43% in the RT group. Participants were required to attend more than 70% of sessions, and missed sessions could be made up. | Completer |
|  | CG |  |  | 31 | 11/20 | 56.29±5.92 | 26.92±1.74 | ＞2 |  |  |  | No | Yes | NR | Not applicable | Completer |
| Balducci 2010 | CE | Italy | ①, ② | 288 | 173/115 | 58.8±8.6 | 31.2±4.6 |  | 52 | 2 | 75 | Yes | Yes | NR | NR | ITT |
|  | CG |  |  | 275 | 156/119 | 58.8±8.5 | 31.9±4.6 |  |  |  |  | Yes | Yes | NR | Not applicable | ITT |
| Alvarez 2016 | HIIT | Chile | ①, ② | 13 | 0/13 | 45.6±11.18 | 30.6±3.97 | 3.4±3.97 | 16 | 3 | 30 | NR | NR | NR | NR | NR |
|  | CG |  |  | 10 | 0/10 | 43.1±4.74 | 30.4±1.26 | 3.6±3.48 |  |  |  | NR | NR | NR | Not applicable | NR |
| Hwang 2019 | HIIT | USA | ①, ② | 18 | 9/9 | 65±8.49 | 31.7±5.52 | 7.8±5.52 | 8 | 4 | 25 | NR | NR | NR | NR | NR |
|  | CAE |  |  | 16 | 10/6 | 62±8.00 | 31.8±5.60 | 8.3±6.00 | 8 | 4 | 30 | NR | NR | NR | NR | NR |
|  | CG |  |  | 16 | 10/6 | 61±8.00 | 33.9±5.60 | 8.2±6.00 |  |  |  | NR | NR | NR | Not applicable | NR |
| Jorge 2011 | CAE | Brazil | ① | 12 | 5/7 | 52.09±8.71 | 29.30±2.09 | 5.45±4.12 | 12 | 3 | 60 | NR | NR | NR | NR | NR |
|  | RT |  |  | 12 | 5/7 | 54.1±8.94 | 31.29±4.08 | 7.70±3.90 | 12 | 3 | 60 | NR | NR | NR | NR | NR |
|  | CE |  |  | 12 | 4/8 | 57.90±9.82 | 31.24±3.88 | 7.30±4.97 | 12 | 3 | 60 | NR | NR | NR | NR | NR |
|  | CG |  |  | 12 | 4/8 | 53.41±9.82 | 30.03±4.90 | 5.25±3.52 |  |  |  | NR | NR | NR | Not applicable | NR |
| Castaneda 2002 | RT | USA | ② | 31 | 10/21 | 66±11.14 | 30.9±6.12 | 8±5.57 | 16 | 3 | 45 | No | No | No | NR | ITT |
|  | CG |  |  | 31 | 12/19 | 66±5.57 | 31.2±5.57 | 11±5.57 |  |  |  | No | No | No | Not applicable | ITT |
| Lambers 2008 | CE | Belgium | ① | 17 | 7/10 | 55.8±9.66 | 28.9±2.84 |  | 12 | 3 | 60 | NR | NR | NR | NR | NR |
|  | CAE |  |  | 18 | 16/2 | 52.2±8.26 | 30.9±4.03 |  | 12 | 3 | 60 | NR | NR | NR | NR | NR |
|  | CG |  |  | 11 | 6/5 | 57.5±8.69 | 30.4±4.32 |  |  |  |  | NR | NR | NR | Not applicable | NR |
| Bacchi 2013 | CAE | Italy | ① | 14 | 10/4 | 55.6±7.48 | 30.5±3.74 | 9.5±6.36 | 16 | 3 | 60 | No | No | No | Partially reported: Exercise adherence was described as good, but a specific attendance percentage was not reported. | NR |
|  | RT |  |  | 17 | 12/5 | 56.0±7.83 | 28.8±4.54 | 9.8±8.25 | 16 | 3 | 60 | No | No | No | Partially reported: Exercise adherence was described as good, but a specific attendance percentage was not reported. | NR |
| Yavari 2012 | CAE | Iran | ①, ② | 15 | NR | 48.2±9.2 | 29.4±5.7 | >1 | 52 | 3 | 40 | No | No | NR | NR | Completer |
|  | RT |  |  | 15 | NR | 51.5±6.3 | 30.3±4.0 | >1 | 52 | 3 | 20 | No | No | NR | NR | Completer |
|  | CE |  |  | 15 | NR | 50.9±9.8 | 28.8±5.4 | >1 | 52 | 3 | 60 | No | No | NR | NR | Completer |
|  | CG |  |  | 15 | NR | 51.5±8.5 | 32.0±4.9 | >1 |  |  |  | No | No | NR | Not applicable | Completer |
| Han Ying 2015 | CAE | China | ① | 32 | 0/32 | 54.1±7.24 | 25.4±1.91 | 8.67±3.29 | 12 | 5 | 60 | NR | NR | NR | NR | NR |
|  | CG |  |  | 30 | 0/30 | 53.7±6.8 | 25.7±2.06 | 7.89±3.27 |  |  |  | NR | NR | NR | Not applicable | NR |
| Chao Min 2015 | CAE | China | ①, ② | 15 | 8/7 | 54±6.53 | 29.54±4.37 | 4±2.98 | 12 | 3 | 55 | NR | NR | NR | NR | NR |
|  | CG |  |  | 15 | 6/9 | 53±7.2 | 29.73±4.26 | 4±3.11 |  |  |  | NR | NR | NR | Not applicable | NR |
| Wu Yanping 2022 | HIIT | China | ①, ② | 23 | 14/9 | 54±7 | 28. 3±2.6 | 7±5 | 12 | 3 | 20 | No | NR | No | NR | NR |
|  | CAE |  |  | 21 | 13/8 | 57±8 | 27. 6±3.2 | 6±4 | 12 | 3 | 45 | No | NR | No | NR | NR |
|  | CG |  |  | 18 | 13/5 | 56±6 | 28. 1±2. 8 | 6±5 |  |  |  | No | NR | No | Not applicable | NR |
| Sabouri 2021 | HIIT | Iran | ①, ② | 16 | 9/7 | 52.02±18.36 | 28.01±10.88 | 7.25±9.52 | 12 | 3 | 30 | NR | NR | No | NR | NR |
|  | RT |  |  | 15 | 7/8 | 51.31±17.31 | 28.69±10.69 | 7.40±9.68 | 12 | 3 | 40 | NR | NR | No | NR | NR |
|  | CG |  |  | 13 | 6/7 | 52.28±11.39 | 26.58±9.30 | 6.76±9.55 |  |  |  | NR | NR | No | Not applicable | NR |
| Moe 2011 | CAE | Norway | ①, ② | 13 | 13/0 | 56.23±8.29 | 30.75±3.34 | 4.69±3.85 | 12 | 3 | 45 | NR | NR | NR | NR | NR |
|  | RT |  |  | 13 | 13/0 | 57.77 ±7.82 | 29.33±4.01 | 4.85±4.25 | 12 | 3 | 45 | NR | NR | NR | NR | NR |
| Abdelbasset 2020 | HIIT | Saudi Arabia | ①, ② | 16 | 10/6 | 54.4±5.8 | 36.3±4.5 |  | 8 | 3 | 25 | NR | NR | NR | NR | NR |
|  | CAE |  |  | 15 | 8/7 | 54.9±4.7 | 36.7±3.4 |  | 8 | 3 | 45 | NR | NR | NR | NR | NR |
|  | CG |  |  | 16 | 9/7 | 55.2±4.3 | 35.9±5.3 |  |  |  |  | NR | NR | NR | Not applicable | NR |
| Zhang Ying 2008 | TCS | China | ①, ② | 10 | 0/10 | 57.4±6.2 |  | 4.4±2.2 | 14 | 5 | 60 | NR | NR | NR | NR | NR |
|  | CG |  |  | 10 | 0/10 | 57.4±6.2 |  | 4.4±2.2 |  |  |  | NR | NR | NR | Not applicable | NR |
| Parra-Sánchez 2015 | CAE | Spain | ①, ② | 47 |  | 73.2±4.8 | 31.3±3.9 |  | 12 | 2 | 40 | NR | NR | NR | NR | ITT |
|  | CG |  |  | 41 |  | 72.6±5.1 | 32.7±4.3 |  |  |  |  | NR | NR | NR | Not applicable | ITT |
| de Oliveira 2013 | CE | Portugal | ①, ② | 12 | 3/9 | 65.7±8.6 | 30.3±4.1 | 6.1±4.5 | 20 | 3 | 60 | NR | NR | NR | Reported: Exercise adherence was greater than 80%, and dropouts were described. | PP |
|  | CG |  |  | 13 | 6/7 | 65.5±5.6 | 30.8±4.3 | 11.3±8.5 |  |  |  | NR | NR | NR | Not applicable | PP |
| Bonfante 2022 | CE | Brazil | ① | 17 | 7/10 | 51.10±3.94 | 29.56±3.56 | 5.5±2.62 | 16 | 3 | 60 | NR | NR | No | NR | NR |
|  | CG |  |  | 17 | 9/8 | 52.41±4.44 | 29.36±3.32 | 4.94±3.05 |  |  |  | NR | NR | No | Not applicable | NR |
| Pardeshi 2024 | Yoga | India | ① | 40 | 40/0 | 40-60 |  |  | 12 | 7 | 60 | NR | NR | NR | NR | NR |
|  | CG |  |  | 38 | 38/0 | 40-60 |  |  |  |  |  | NR | NR | NR | Not applicable | NR |

Note：CAE, continuous aerobic exercise; CE, combined aerobic and resistance exercise; RT, resistance training; TCS, traditional Chinese sports. USA, the United States of America. NR, not reported. ITT, intention-to-treat analysis; PP, per-protocol analysis. ①:TG; ②:LDL-C.

4. **Risk of bias**

|  | **Bias arising from the randomization process** | **Bias due to deviations from intended interventions (effect of assignment to intervention)** | **Bias due to missing outcome data** | **Bias in measurement of the outcome** | **Bias in selection of the reported result** | **Overall bias** |
| --- | --- | --- | --- | --- | --- | --- |
| Wu Meiqiao 2020 | 2 | 2 | 1 | 1 | 1 | 2 |
| Arora 2007 | 3 | 1 | 2 | 1 | 2 | 3 |
| Sigal 2007 | 2 | 2 | 1 | 1 | 1 | 2 |
| Honkola 1997 | 2 | 2 | 1 | 1 | 1 | 2 |
| Cauza 2006 | 1 | 2 | 1 | 1 | 1 | 2 |
| Keun Hee An 2005 | 1 | 2 | 1 | 1 | 1 | 2 |
| Cauza 2005 | 1 | 2 | 1 | 1 | 1 | 2 |
| Kadoglou 2012 | 1 | 1 | 1 | 1 | 1 | 1 |
| Kadoglou NP 2012 | 1 | 1 | 1 | 1 | 1 | 1 |
| Pandey 2017 | 1 | 1 | 1 | 1 | 1 | 1 |
| Koh 2018 | 1 | 2 | 1 | 1 | 1 | 2 |
| Dede 2015 | 2 | 2 | 1 | 1 | 1 | 2 |
| Rahbar 2017 | 1 | 2 | 1 | 1 | 1 | 2 |
| Liu Shuxiu 2017 | 2 | 3 | 1 | 2 | 1 | 3 |
| Choi 2012 | 1 | 1 | 1 | 1 | 1 | 1 |
| Tan Sijie 2012 | 2 | 1 | 1 | 1 | 1 | 2 |
| Nagarathna 2012 | 1 | 1 | 1 | 1 | 1 | 1 |
| Yang Dan 2020 | 2 | 2 | 1 | 1 | 1 | 2 |
| Kim 2006 | 2 | 2 | 1 | 1 | 1 | 2 |
| Li Songqian 2021 | 2 | 2 | 2 | 1 | 1 | 2 |
| Li Kelei 2020 | 2 | 2 | 2 | 1 | 1 | 2 |
| Zhao Gang 2017 | 2 | 2 | 1 | 1 | 1 | 2 |
| Meng En 2014 | 2 | 2 | 2 | 1 | 1 | 2 |
| Jiang Rongkang 2007 | 2 | 2 | 2 | 1 | 2 | 2 |
| Zhu Yunfei 2023 | 2 | 2 | 1 | 1 | 1 | 2 |
| Li Xiaoxiao 2023 | 1 | 2 | 2 | 1 | 1 | 2 |
| Qi Yonghao 2023 | 2 | 2 | 1 | 1 | 1 | 2 |
| Zhao Tianqi 2022 | 2 | 2 | 2 | 1 | 1 | 2 |
| He Ke 2019 | 2 | 2 | 1 | 1 | 1 | 2 |
| Guo Qianqian 2019 | 2 | 2 | 2 | 1 | 2 | 2 |
| Li Yue 2017 | 1 | 2 | 2 | 1 | 1 | 2 |
| Wang Chengyuan 2015 | 1 | 1 | 1 | 1 | 1 | 1 |
| Xu Yuxin 2019 | 2 | 2 | 1 | 1 | 1 | 2 |
| Kan Wei 2021 | 2 | 2 | 2 | 1 | 3 | 3 |
| Lu Fulin 2018 | 2 | 2 | 1 | 1 | 1 | 2 |
| Xu Lianwu 2013 | 1 | 1 | 1 | 1 | 1 | 1 |
| Wang Ping 2019 | 3 | 2 | 1 | 1 | 1 | 3 |
| Farias 2015 | 1 | 2 | 1 | 1 | 1 | 2 |
| Gordon 2008 | 1 | 2 | 2 | 2 | 1 | 2 |
| Singh 2008 | 2 | 2 | 1 | 1 | 1 | 2 |
| Huang Rongchun 2011 | 2 | 2 | 1 | 1 | 1 | 2 |
| Zhang Jie 2015 | 1 | 1 | 1 | 1 | 1 | 1 |
| Chang Feng 2018 | 2 | 2 | 2 | 1 | 1 | 2 |
| Hordern 2008 | 2 | 2 | 2 | 1 | 1 | 2 |
| Praet 2008 | 1 | 1 | 1 | 1 | 1 | 1 |
| Farzanegi 2017 | 1 | 2 | 1 | 1 | 1 | 2 |
| Barone Gibbs 2012 | 2 | 2 | 1 | 1 | 1 | 2 |
| Kazemi 2024 | 2 | 2 | 2 | 1 | 1 | 2 |
| Zadeh 2022 | 2 | 1 | 1 | 2 | 1 | 2 |
| Al Ozairi 2023 | 1 | 2 | 2 | 1 | 1 | 2 |
| Ma Xiaojun 2024 | 2 | 2 | 2 | 1 | 1 | 2 |
| Balducci 2010 | 1 | 1 | 1 | 1 | 1 | 1 |
| Alvarez 2016 | 2 | 2 | 2 | 1 | 1 | 2 |
| Hwang 2019 | 2 | 2 | 2 | 1 | 1 | 2 |
| Jorge 2011 | 2 | 2 | 2 | 1 | 1 | 2 |
| Castaneda 2002 | 2 | 2 | 1 | 1 | 1 | 2 |
| Lambers 2008 | 2 | 2 | 2 | 1 | 1 | 2 |
| Bacchi 2013 | 2 | 2 | 2 | 1 | 1 | 2 |
| Yavari 2012 | 2 | 2 | 3 | 1 | 1 | 3 |
| Han Ying 2015 | 2 | 2 | 2 | 1 | 1 | 2 |
| Chao Min 2015 | 2 | 2 | 2 | 1 | 1 | 2 |
| Wu Yanping 2022 | 2 | 1 | 1 | 1 | 1 | 2 |
| Sabouri 2021 | 2 | 2 | 2 | 1 | 1 | 2 |
| Moe 2011 | 2 | 2 | 2 | 1 | 1 | 2 |
| Abdelbasset 2020 | 1 | 1 | 1 | 1 | 1 | 1 |
| Zhang Ying 2008 | 2 | 2 | 2 | 1 | 2 | 2 |
| Parra-Sánchez 2015 | 1 | 2 | 1 | 1 | 1 | 2 |
| de Oliveira 2013 | 2 | 2 | 2 | 1 | 1 | 2 |
| Bonfante 2022 | 2 | 2 | 2 | 1 | 1 | 2 |
| Pardeshi 2024 | 2 | 2 | 2 | 1 | 1 | 2 |

Note: 1, Low risk；2, Some concerns；3, High risk.

**5. Confidence of evidence**

The confidence in the evidence for the network meta-analysis was assessed using the Confidence in Network Meta-Analysis (CINeMA) framework. For each lipid outcome, including triglycerides (TG) and low-density lipoprotein cholesterol (LDL-C), we assessed the confidence in the estimated effects of each exercise modality compared with the control group. The exercise modalities included continuous aerobic exercise (CAE), combined aerobic and resistance exercise (CE), traditional Chinese exercise (TCS), resistance training (RT), high-intensity interval training (HIIT), and yoga.

In line with the CINeMA approach, six domains were evaluated: within-study bias, reporting bias, indirectness, imprecision, heterogeneity, and incoherence. Similar to the GRADE approach, the confidence in evidence was initially rated as high and was then downgraded according to the level of concern in each domain. The final confidence rating was classified as high, moderate, low, or very low.

The overall confidence rating for each exercise modality compared with the control group was determined according to the level of concern across the six CINeMA domains.

**Table S1.** Summary table of confidence of evidence for TG

| **Comparison** | **Number of studies** | **Within-study bias** | **Reporting bias** | **Indirectness** | **Imprecision** | **Heterogeneity** | **Incoherence** | **Confidence rating** |
| --- | --- | --- | --- | --- | --- | --- | --- | --- |
| CAE | 29 | Some concerns | Low risk | Some concerns | No concerns | Some concerns | No concerns | Low |
| CE | 13 | Some concerns | Low risk | Some concerns | No concerns | Some concerns | No concerns | Low |
| HIIT | 6 | Some concerns | Some concerns | Some concerns | No concerns | No concerns | No concerns | Low |
| RT | 11 | Some concerns | Low risk | No concerns | No concerns | Some concerns | No concerns | Moderate |
| TCS | 15 | Some concerns | Low risk | Some concerns | No concerns | Some concerns | No concerns | Low |
| Yoga | 3 | Some concerns | Some concerns | Some concerns | No concerns | Some concerns | No concerns | Low |

**Table S2.** Summary table of confidence of evidence for LDL-C

| **Comparison** | **Number of studies** | **Within-study bias** | **Reporting bias** | **Indirectness** | **Imprecision** | **Heterogeneity** | **Incoherence** | **Confidence rating** |
| --- | --- | --- | --- | --- | --- | --- | --- | --- |
| CAE | 23 | Some concerns | Low risk | Some concerns | No concerns | Some concerns | No concerns | Low |
| CE | 10 | Some concerns | Low risk | Some concerns | No concerns | No concerns | No concerns | Moderate |
| HIIT | 6 | Some concerns | Some concerns | Some concerns | No concerns | Some concerns | Some concerns | Low |
| RT | 10 | Some concerns | Low risk | Some concerns | No concerns | Some concerns | No concerns | Low |
| TCS | 15 | Some concerns | Low risk | Some concerns | No concerns | Some concerns | Some concerns | Low |
| Yoga | 2 | Some concerns | Some concerns | No concerns | No concerns | Some concerns | No concerns | Low |

**6. Pairwise meta-analysis and publication bias**

**6.1 Details of pairwise meta-analysis**

**Table S3.** Details of pairwise meta-analysis for TG

| **Comparison** | **Number of studies** | **MD** | **95% CrI** | **I^2^** |
| --- | --- | --- | --- | --- |
| CAE vs CG | 29 | **-0.29** | **(-0.40, -0.17)** | 59.1 |
| CE vs CG | 13 | **-0.23** | **(-0.46, -0.03)** | 38.6 |
| HIIT vs CG | 6 | **-0.3** | **(-0.60, -0.07)** | 27.7 |
| RT vs CG | 11 | **-0.26** | **(-0.47, -0.07)** | 17.5 |
| TCS vs CG | 15 | **-0.38** | **(-0.58, -0.19)** | 84.7 |
| Yoga vs CG | 3 | -0.41 | (-1.04, 0.24) | 60.7 |

**Table S4.** Details of pairwise meta-analysis for LDL-C

| **Comparison** | **Number of studies** | **MD** | **95% CrI** | **I^2^** |
| --- | --- | --- | --- | --- |
| CAE vs CG | 23 | **-0.37** | **(-0.50, -0.25)** | 56.2 |
| CE vs CG | 10 | **-0.56** | **(-1.00, -0.15)** | 91 |
| HIIT vs CG | 6 | -0.13 | (-0.36, 0.11) | 17.5 |
| RT vs CG | 10 | **-0.3** | **(-0.51, -0.09)** | 51.5 |
| TCS vs CG | 15 | **-0.31** | **(-0.42, -0.21)** | 35.1 |
| Yoga vs CG | 2 | -0.27 | (-0.93, 0.39) | 41.6 |

***Note:*** Numbers in bold indicate significant results. MD: mean difference; CrI: credible interval.

CAE, continuous aerobic exercise; CE, combined aerobic and resistance exercise; RT, resistance training; TCS, traditional Chinese sports.

**6.2 Publication bias**

**Figure S1.** Funnel plot of publication bias for TG

**Figure S2.** Funnel plot of publication bias for LDL-C

***Note:*** CAE, continuous aerobic exercise; CE, combined aerobic and resistance exercise; RT, resistance training; TCS, traditional Chinese sports.

**7. Bayesian network meta-analysis**

**7.1 Model fit summaries**

**Table S5.** Model fit summaries for all included studies

| **Model** | **TG (158 data points)** | | | |
| --- | --- | --- | --- | --- |
|  | **DIC** | **pD** | **Residual Deviance** | **SD (95% CrI)** |
| FE Model | 376.1 | 74 | 302.1 | - |
| RE Model | 261.7 | 114.3 | 147.4 | 0.21 (0.16, 0.28) |
| RE UME | 264.8 | 117.6 | 147.1 | - |
| **Model** | **LDL-C (140 data points)** | | | |
|  | **DIC** | **pD** | **Residual Deviance** | **SD (95% CrI)** |
| FE Model | 325.4 | 67 | 258.4 | - |
| RE Model | 250.8 | 106.9 | 143.9 | 0.23 (0.16, 0.31) |
| RE UME | 254.2 | 110.4 | 143.8 | - |

***Note:*** FE: fixed effects; RE: random effects; UME: unrelated mean effects; DIC: deviance information criterion; pD: number of effective parameters; SD: between-study standard deviation; CrI: credible interval.

**7.2 Node-split method for assessment of inconsistency**

**Table S6.** Results of node-split method for TG

| **Comparison** | **p-value** | **MD (95% CrI)** |
| --- | --- | --- |

| d.CAE.CE | 0.262 |  |
| --- | --- | --- |
| -> direct |  | -0.12 (-0.36, 0.12) |
| -> indirect |  | 0.076 (-0.19, 0.34) |
| -> network |  | -0.032 (-0.20, 0.14) |
| d.CAE.CG | 0.5024 |  |
| -> direct |  | 0.29 (0.17, 0.41) |
| -> indirect |  | 0.19 (-0.056, 0.46) |
| -> network |  | 0.27 (0.17, 0.37) |
| d.CAE.HIIT | 0.179 |  |
| -> direct |  | -0.29 (-0.51, -0.066) |
| -> indirect |  | 0.0017 (-0.37, 0.35) |
| -> network |  | -0.21 (-0.38, -0.026) |
| d.CAE.RT | 0.8222 |  |
| -> direct |  | -0.020 (-0.22, 0.18) |
| -> indirect |  | 0.022 (-0.30, 0.36) |
| -> network |  | -0.011 (-0.17, 0.15) |
| d.CAE.TCS | 0.1672 |  |
| -> direct |  | 0.17 (-0.21, 0.54) |
| -> indirect |  | -0.12 (-0.31, 0.056) |
| -> network |  | -0.070 (-0.23, 0.093) |
| d.CAE.Yoga | 0.5682 |  |
| -> direct |  | 0.028 (-0.45, 0.51) |
| -> indirect |  | -0.14 (-0.53, 0.23) |
| -> network |  | -0.078 (-0.38, 0.22) |
| d.CE.CG | 0.5748 |  |
| -> direct |  | 0.27 (0.081, 0.46) |
| -> indirect |  | 0.39 (0.0043, 0.76) |
| -> network |  | 0.30 (0.14, 0.47) |
| d.CE.HIIT | 0.9726 |  |
| -> direct |  | -0.18 (-0.75, 0.38) |
| -> indirect |  | -0.17 (-0.43, 0.093) |
| -> network |  | -0.17 (-0.41, 0.059) |
| d.CE.RT | 0.2968 |  |
| -> direct |  | 0.19 (-0.19, 0.57) |
| -> indirect |  | -0.050 (-0.31, 0.21) |
| -> network |  | 0.020 (-0.18, 0.23) |
| d.CE.TCS | 0.7506 |  |
| -> direct |  | 0.097 (-0.74, 0.96) |
| -> indirect |  | -0.048 (-0.26, 0.17) |
| -> network |  | -0.039 (-0.25, 0.17) |
| d.CG.HIIT | 0.0764 |  |
| -> direct |  | -0.35 (-0.61, -0.080) |
| -> indirect |  | -0.69 (-0.97, -0.43) |
| -> network |  | -0.48 (-0.66, -0.30) |
| d.CG.RT | 0.7084 |  |
| -> direct |  | -0.31 (-0.52, -0.12) |
| -> indirect |  | -0.24 (-0.60, 0.11) |
| -> network |  | -0.28 (-0.44, -0.12) |
| d.CG.TCS | 0.1358 |  |
| -> direct |  | -0.38 (-0.52, -0.24) |
| -> indirect |  | 0.11 (-0.51, 0.71) |
| -> network |  | -0.34 (-0.48, -0.21) |
| d.CG.Yoga | 0.5744 |  |
| -> direct |  | -0.41 (-0.78, -0.036) |
| -> indirect |  | -0.23 (-0.73, 0.27) |
| -> network |  | -0.35 (-0.64, -0.049) |
| d.HIIT.RT | 0.8294 |  |
| -> direct |  | 0.16 (-0.28, 0.59) |
| -> indirect |  | 0.21 (-0.048, 0.47) |
| -> network |  | 0.19 (-0.024, 0.42) |

**Table S7.** Results of node-split method for LDL-C

| **Comparison** | **p-value** | **MD (95% CrI)** |
| --- | --- | --- |

| d.CAE.CE | 0.7005 |  |
| --- | --- | --- |
| -> direct |  | -0.13 (-0.38, 0.11) |
| -> indirect |  | -0.065 (-0.34, 0.18) |
| -> network |  | -0.11 (-0.30, 0.066) |
| d.CAE.CG | 0.56625 |  |
| -> direct |  | 0.37 (0.26, 0.48) |
| -> indirect |  | 0.30 (0.076, 0.52) |
| -> network |  | 0.35 (0.24, 0.46) |
| d.CAE.HIIT | 0.25325 |  |
| -> direct |  | -0.067 (-0.28, 0.15) |
| -> indirect |  | 0.21 (-0.20, 0.63) |
| -> network |  | 0.017 (-0.17, 0.21) |
| d.CAE.RT | 0.86375 |  |
| -> direct |  | 0.058 (-0.14, 0.27) |
| -> indirect |  | 0.024 (-0.32, 0.37) |
| -> network |  | 0.032 (-0.13, 0.20) |
| d.CAE.TCS | 0.8085 |  |
| -> direct |  | 0.11 (-0.29, 0.50) |
| -> indirect |  | 0.049 (-0.15, 0.25) |
| -> network |  | 0.059 (-0.11, 0.23) |
| d.CAE.Yoga | 0.5475 |  |
| -> direct |  | -0.18 (-0.56, 0.20) |
| -> indirect |  | 0.080 (-0.68, 0.82) |
| -> network |  | -0.099 (-0.42, 0.24) |
| d.CE.CG | 0.1565 |  |
| -> direct |  | 0.49 (0.29, 0.70) |
| -> indirect |  | 0.19 (-0.18, 0.56) |
| -> network |  | 0.46 (0.29, 0.64) |
| d.CE.HIIT | 0.20525 |  |
| -> direct |  | -0.18 (-0.72, 0.37) |
| -> indirect |  | 0.20 (-0.060, 0.49) |
| -> network |  | 0.13 (-0.10, 0.38) |
| d.CE.RT | 0.7 |  |
| -> direct |  | 0.089 (-0.29, 0.49) |
| -> indirect |  | 0.18 (-0.084, 0.47) |
| -> network |  | 0.15 (-0.064, 0.37) |
| d.CE.TCS | 0.0045 |  |
| -> direct |  | 1.1 (0.44, 1.8) |
| -> indirect |  | 0.061 (-0.15, 0.28) |
| -> network |  | 0.17 (-0.041, 0.40) |
| d.CG.HIIT | 0.05 |  |
| -> direct |  | -0.12 (-0.38, 0.15) |
| -> indirect |  | -0.52 (-0.80, -0.22) |
| -> network |  | -0.33 (-0.52, -0.14) |
| d.CG.RT | 0.623 |  |
| -> direct |  | -0.30 (-0.50, -0.088) |
| -> indirect |  | -0.40 (-0.74, -0.060) |
| -> network |  | -0.31 (-0.47, -0.15) |
| d.CG.TCS | 0.4055 |  |
| -> direct |  | -0.32 (-0.46, -0.19) |
| -> indirect |  | -0.050 (-0.69, 0.56) |
| -> network |  | -0.29 (-0.43, -0.15) |
| d.CG.Yoga | 0.303 |  |
| -> direct |  | -0.27 (-0.73, 0.19) |
| -> indirect |  | -0.63 (-1.1, -0.11) |
| -> network |  | -0.45 (-0.77, -0.11) |
| d.HIIT.RT | 0.90225 |  |
| -> direct |  | 0.030 (-0.48, 0.53) |
| -> indirect |  | -0.0020 (-0.26, 0.27) |
| -> network |  | 0.013 (-0.22, 0.24) |

***Note:*** MD: mean difference; CrI: credible interval; CAE, continuous aerobic exercise; CE, combined aerobic and resistance exercise; RT, resistance training; TCS, traditional Chinese sports.

**7.3 Forest plots showing the effects of exercise modality on blood lipid levels**

a

b

**Figure S3.** Forest plots showing the effects of exercise modality on TG (a) and LDL-C (b)

***Note:*** Results are presented in mean difference. CrI: credible interval; CAE, continuous aerobic exercise; CE, combined aerobic and resistance exercise; RT, resistance training; TCS, traditional Chinese sports.

**7.4 SUCRA table for all studies**

**Table S8.** SUCRA table of TG

| **Treatment** | **Rank 1** | **Rank 2** | **Rank 3** | **Rank 4** | **Rank 5** | **Rank 6** | **Rank 7** | **SUCRA** |
| --- | --- | --- | --- | --- | --- | --- | --- | --- |
| CAE | 0.001 | 0.026 | 0.114 | 0.268 | 0.356 | 0.236 | 0 | 39.00 |
| CE | 0.034 | 0.15 | 0.22 | 0.216 | 0.187 | 0.193 | 0 | 50.79 |
| CG | 0 | 0 | 0 | 0 | 0 | 0.012 | 0.988 | 0.20 |
| HIIT | 0.688 | 0.222 | 0.058 | 0.021 | 0.009 | 0.002 | 0 | 92.53 |
| RT | 0.013 | 0.099 | 0.175 | 0.218 | 0.23 | 0.265 | 0 | 44.18 |
| TCS | 0.065 | 0.263 | 0.293 | 0.18 | 0.12 | 0.08 | 0 | 62.25 |
| Yoga | 0.2 | 0.241 | 0.139 | 0.098 | 0.099 | 0.212 | 0.012 | 61.05 |

**Table S9.** SUCRA table of LDL-C

| **Treatment** | **Rank 1** | **Rank 2** | **Rank 3** | **Rank 4** | **Rank 5** | **Rank 6** | **Rank 7** | **SUCRA** |
| --- | --- | --- | --- | --- | --- | --- | --- | --- |
| CAE | 0.016 | 0.128 | 0.328 | 0.313 | 0.167 | 0.048 | 0 | 56.15 |
| CE | 0.443 | 0.369 | 0.102 | 0.051 | 0.025 | 0.009 | 0 | 85.45 |
| CG | 0 | 0 | 0 | 0 | 0 | 0.005 | 0.995 | 0.08 |
| HIIT | 0.054 | 0.147 | 0.188 | 0.181 | 0.211 | 0.219 | 0.001 | 49.90 |
| RT | 0.026 | 0.097 | 0.168 | 0.214 | 0.253 | 0.242 | 0 | 45.01 |
| TCS | 0.014 | 0.063 | 0.123 | 0.168 | 0.266 | 0.366 | 0 | 38.19 |
| Yoga | 0.447 | 0.197 | 0.091 | 0.073 | 0.078 | 0.111 | 0.004 | 75.20 |

***Note:*** CAE, continuous aerobic exercise; CE, combined aerobic and resistance exercise; RT, resistance training; TCS, traditional Chinese sports.

**7.5 Assessment of transitivity assumption**

TG

LDL-C

Figure S4. Distribution of potential effect modifiers across intervention nodes. The four panels illustrate the distribution of age, baseline lipid level, intervention duration in weeks, and sample size across intervention nodes, providing a visual assessment of comparability across the treatment network.

**7.6 Sensitivity analysis**

7.6.1 Forest plots and SUCRA ranking plots of sensitivity analysis

a. Exclude high risk of bias

b. Exclude small sample size

**Figure S5.** Forest plots and SUCRA ranking plots of sensitivity analysis for TG

***Note:*** Results are presented in mean difference. CrI: credible interval; CAE, continuous aerobic exercise; CE, combined aerobic and resistance exercise; RT, resistance training; TCS, traditional Chinese sports. Small-sample studies were defined as trials with fewer than 10 participants in at least one study arm.

a. Exclude high risk of bias

b. Exclude small sample size

**Figure S6.** Forest plots and SUCRA ranking plots of sensitivity analysis for LDL-C

***Note:*** Results are presented in mean difference. CrI: credible interval; CAE, continuous aerobic exercise; CE, combined aerobic and resistance exercise; RT, resistance training; TCS, traditional Chinese sports.

7.6.2 League tables of sensitivity analysis

**Table S11.** League tables of sensitivity analysis for TG

| **a. Exclude high risk of bias** | | | | | | |
| --- | --- | --- | --- | --- | --- | --- |
| CAE |  |  |  |  |  |  |
| 0.01(-0.16, 0.19) | CE |  |  |  |  |  |
| 0.21(0.03, 0.39) | 0.20(-0.03, 0.43) | HIIT |  |  |  |  |
| 0.00(-0.17, 0.18) | -0.01(-0.23, 0.21) | -0.21(-0.42, 0.02) | RT |  |  |  |
| 0.11(-0.07, 0.28) | 0.09(-0.12, 0.31) | -0.11(-0.34, 0.12) | 0.10(-0.11, 0.32) | TCS |  |  |
| 0.08(-0.22, 0.39) | 0.07(-0.26, 0.40) | -0.13(-0.47, 0.22) | 0.08(-0.26, 0.42) | -0.02(-0.34, 0.30) | Yoga |  |
| -0.25(-0.36, -0.15) | -0.27(-0.43, -0.10) | -0.47(-0.65, -0.28) | -0.26(-0.43, -0.09) | -0.36(-0.50, -0.22) | -0.34(-0.64, -0.05) | CG |
| **b. Exclude small sample size** | | | | | | |
| CAE |  |  |  |  |  |  |
| 0.04(-0.14, 0.21) | CE |  |  |  |  |  |
| 0.21(0.03, 0.40) | 0.18(-0.07, 0.41) | HIIT |  |  |  |  |
| 0.02(-0.14, 0.20) | -0.01(-0.23, 0.21) | -0.19(-0.42, 0.04) | RT |  |  |  |
| 0.07(-0.10, 0.23) | 0.03(-0.18, 0.24) | -0.15(-0.38, 0.09) | 0.04(-0.18, 0.26) | TCS |  |  |
| 0.07(-0.24, 0.38) | 0.04(-0.31, 0.38) | -0.14(-0.49, 0.21) | 0.05(-0.29, 0.39) | 0.01(-0.33, 0.35) | Yoga |  |
| -0.27(-0.37, -0.17) | -0.30(-0.47, -0.15) | -0.48(-0.67, -0.29) | -0.29(-0.47, -0.13) | -0.34(-0.48, -0.20) | -0.34(-0.65, -0.04) | CG |

***Note:*** Results are presented in mean difference (95% CrI). CAE, continuous aerobic exercise; CE, combined aerobic and resistance exercise; RT, resistance training; TCS, traditional Chinese sports.

**Table S12.** League tables of sensitivity analysis for LDL-C

| **a. Exclude high risk of bias** | | | | | | |
| --- | --- | --- | --- | --- | --- | --- |
| CAE |  |  |  |  |  |  |
| 0.11(-0.08, 0.30) | CE |  |  |  |  |  |
| -0.01(-0.20, 0.17) | -0.12(-0.37, 0.12) | HIIT |  |  |  |  |
| -0.00(-0.18, 0.16) | -0.11(-0.34, 0.11) | 0.01(-0.22, 0.24) | RT |  |  |  |
| -0.04(-0.22, 0.14) | -0.14(-0.38, 0.07) | -0.02(-0.26, 0.22) | -0.03(-0.26, 0.18) | TCS |  |  |
| 0.10(-0.22, 0.43) | -0.01(-0.38, 0.35) | 0.12(-0.25, 0.48) | 0.11(-0.25, 0.46) | 0.14(-0.22, 0.50) | Yoga |  |
| -0.33(-0.45, -0.22) | -0.44(-0.62, -0.27) | -0.32(-0.51, -0.13) | -0.33(-0.50, -0.16) | -0.30(-0.44, -0.15) | -0.43 (-0.77, -0.11) | CG |
| **b. Exclude small sample size** | | | | | | |
| CAE |  |  |  |  |  |  |
| 0.12(-0.06, 0.31) | CE |  |  |  |  |  |
| -0.01(-0.20, 0.17) | -0.13(-0.39, 0.10) | HIIT |  |  |  |  |
| -0.03(-0.21, 0.14) | -0.15(-0.39, 0.07) | -0.02(-0.26, 0.23) | RT |  |  |  |
| -0.05(-0.23, 0.12) | -0.18(-0.41, 0.03) | -0.04(-0.28, 0.19) | -0.02(-0.25, 0.20) | TCS |  |  |
| 0.10(-0.22, 0.42) | -0.02(-0.40, 0.34) | 0.11(-0.26, 0.48) | 0.14(-0.23, 0.49) | 0.15(-0.20, 0.51) | Yoga |  |
| -0.33(-0.45, -0.22) | -0.45(-0.64, -0.29) | -0.32(-0.51, -0.12) | -0.30(-0.47, -0.12) | -0.28(-0.42, -0.13) | -0.43(-0.75, -0.10) | CG |

***Note:*** Results are presented in mean difference (95% CrI). CAE, continuous aerobic exercise; CE, combined aerobic and resistance exercise; RT, resistance training; TCS, traditional Chinese sports.

7.6.3 Subgroup analysis

Additional subgroup network meta-analyses were performed according to study-level mean age (<60 vs. ≥60 years) and BMI (<30 vs. ≥30 kg/m²). For trials in which baseline age or BMI differed across treatment arms, a sample-size-weighted mean across eligible arms was used to classify the entire trial into a single subgroup. Studies with missing BMI information were excluded from BMI-stratified analyses. All subgroup analyses used the same Bayesian random-effects framework as the primary network meta-analysis.

TG

LDL-C

**Figure S7.** Subgroup network meta-analyses of exercise interventions on TG and LDL-C according to age and BMI.

***Note:*** Results are presented in mean difference. CrI: credible interval; CAE, continuous aerobic exercise; CE, combined aerobic and resistance exercise; RT, resistance training; TCS, traditional Chinese sports.

**7.7 Network meta-regressions**

Network meta-regressions were performed to investigate potential moderating variables and the degree of model fit. The network meta-regression model was fitted in a Bayesian framework using Markov chain Monte Carlo (MCMC) simulation in R package *gemtc*. Four chains were run using a non-informative prior. The number of iterations for each chain was 25,000, with a burn-in period of 5,000 iterations. Convergence was assessed by visual inspection of trajectory plots and potential scaling factors.

**Table S13.** Network meta-regressions

| **Covariate** | **TG (158 data points)** | | | | |
| --- | --- | --- | --- | --- | --- |
|  | **DIC** | **pD** | **Residual**  **Deviance** | **Shared beta**  **(Median and 95% CrI)** | **SD** |
| Unadjusted | 261.7 | 114.3 | 147.4 | - | 0.21 (0.16, 0.28) |
| Age | 262.6 | 115.1 | 147.5 | -0.01 (-0.17, 0.15) | 0.22 (0.16, 0.28) |
| Baseline | 260 | 112.9 | 147.1 | -0.18 (-0.32, -0.03) | 0.20 (0.15, 0.27) |
| Intervention period, weeks | 260.5 | 114.9 | 145.6 | -0.10 (-0.26, 0.05) | 0.22 (0.17, 0.28) |
| Frequency | 260.9 | 113.7 | 147.3 | -0.13 (-0.26, 0.01) | 0.21 (0.16, 0.27) |
| Publication year | 262.8 | 115.1 | 147.7 | 0.01 (-0.17, 0.18) | 0.22 (0.16, 0.28) |
| BMI | 219.1 | 95.6 | 123.5 | 0.02 (-0.15, 0.19) | 0.23 (0.17, 0.31) |
| Percentage of males | 238.5 | 105 | 133.5 | 0.01 (-0.16, 0.17) | 0.23 (0.17, 0.30) |
| Sample Size | 261.9 | 114.5 | 147.4 | 0.06 (-0.07, 0.20) | 0.21 (0.16, 0.28) |
| **Covariate** | **LDL-C (140 data points)** | | | | |
|  | **DIC** | **pD** | **Residual**  **Deviance** | **Shared beta**  **(Median and 95% CrI)** | **SD** |
| Unadjusted | 250.8 | 106.9 | 143.9 | - | 0.23 (0.16, 0.31) |
| Age | 251.1 | 105.6 | 145.5 | -0.16 (-0.33, 0.02) | 0.21 (0.15, 0.29) |
| Baseline | 248.9 | 104.6 | 144.3 | -0.23 (-0.41, -0.06) | 0.21 (0.14, 0.29) |
| Intervention period, weeks | 250.8 | 107.2 | 143.6 | -0.05 (-0.21, 0.11) | 0.23 (0.16, 0.31) |
| Frequency | 249.8 | 105.9 | 144 | -0.19 (-0.38, 0.01) | 0.22 (0.15, 0.30) |
| Publication year | 250.6 | 107.6 | 143.1 | -0.03 (-0.21, 0.14) | 0.23 (0.16, 0.31) |
| BMI | 200.1 | 87.9 | 112.2 | 0.21 (0.02, 0.41) | 0.25 (0.18, 0.35) |
| Percentage of males | 232.4 | 100.6 | 131.8 | 0.05 (-0.12, 0.23) | 0.24 (0.17, 0.32) |
| Sample Size | 249.7 | 107.1 | 142.6 | 0.10 (-0.05, 0.25) | 0.23 (0.16, 0.31) |

***Note:*** CrI, credible interval. BMI, body mass index. DIC, deviance information criterion. SD, standard deviation.

**8. Dose-response network meta-analysis**

**8.1 Key assumptions for network meta-analysis**

8.1.1 Network connectivity

Technically, each treatment of interest must be represented by at least one study and the network needs to be connected. We examined network connectivity at both agent (exercise modality) and treatment (combination of modality and dose) levels and did not find evidence of disconnected network.

**Figure S8.** Treatment-level network (a, TG; b, LDL-C)

a

b

***Note:*** The label indicates the specific exercise modality followed by corresponding exercise dose of that modality. Placebo: control group; CAE, continuous aerobic exercise; CE, combined aerobic and resistance exercise; RT, resistance training; TCS, traditional Chinese sports.

**Figure S9.** Agent-level network (a, TG; b, LDL-C)

a

**b**

***Note:*** Placebo: control group; CAE, continuous aerobic exercise; CE, combined aerobic and resistance exercise; RT, resistance training; TCS, traditional Chinese sports.

8.1.2 Data consistency

To check for consistency at the treatment level using uncorrelated mean effects model (UME), we fit an NMA that does not assume consistency relationships, and that only models the direct

relative effects between each arm in a study and the study reference treatment.90 If the

consistency assumption holds true, then the results from the UME model and the NMA will be very similar.

**Table S14.** NMA and UME model fit comparisons

| **Model** | **TG** | | | | |
| --- | --- | --- | --- | --- | --- |
|  | **pD** | **Deviance** | **Residual deviance** | **DIC** | **SD** |
| NMA | 105.8 | -154.495 | 147.360 | -49.0 | 0.162 |
| UME | 109.0 | -153.709 | 148.146 | -46.0 | 0.158 |
| **Model** | **LDL-C** | | | | |
|  | **pD** | **Deviance** | **Residual deviance** | **DIC** | **SD** |
| NMA | 102.0 | -152.391 | 145.238 | -51.0 | 0.193 |
| UME | 103.6 | -154.282 | 143.348 | -51.8 | 0.180 |

8.1.3 Network transitivity

The assumption of transitivity that indirect comparison validly estimates the unobserved head-to head comparison is a prerequisite to calculate a valid mixed estimate.^90^ We assessed the transitivity by using the MBNMA node-splitting method that splits the contribution of a specific treatment into direct and indirect evidence.

**Table S15.** Node-splitting method of network transitivity for TG

| **Comparison** | **p-value** | **Median** | **2.50%** | **97.50%** |
| --- | --- | --- | --- | --- |
| TCS_750 vs CAE_1000 | 0.188 |  |  |  |
| -> direct |  | 0.357 | -0.168 | 0.911 |
| -> indirect |  | -0.028 | -0.161 | 0.102 |
| -> MBNMA |  | -0.004 | -0.139 | 0.129 |
| HIIT_1000 vs CAE_1000 | 0.25 |  |  |  |
| -> direct |  | -0.649 | -0.994 | -0.304 |
| -> indirect |  | -0.296 | -0.556 | -0.042 |
| -> MBNMA |  | -0.424 | -0.634 | -0.207 |
| CE_1000 vs CAE_1000 | 0.546 |  |  |  |
| -> direct |  | -0.074 | -0.583 | 0.453 |
| -> indirect |  | -0.027 | -0.21 | 0.165 |
| -> MBNMA |  | -0.03 | -0.212 | 0.141 |
| Yoga_750 vs CAE_750 | 0.54 |  |  |  |
| -> direct |  | 0.033 | -0.365 | 0.415 |
| -> indirect |  | -0.182 | -0.494 | 0.104 |
| -> MBNMA |  | -0.106 | -0.348 | 0.133 |
| RT_500 vs CAE_750 | 0.746 |  |  |  |
| -> direct |  | 0.012 | -0.211 | 0.266 |
| -> indirect |  | -0.05 | -0.224 | 0.13 |
| -> MBNMA |  | -0.039 | -0.179 | 0.095 |
| RT_250 vs CAE_750 | 0.237 |  |  |  |
| -> direct |  | -0.125 | -0.65 | 0.383 |
| -> indirect |  | 0.107 | 0.019 | 0.195 |
| -> MBNMA |  | 0.102 | 0.016 | 0.185 |
| HIIT_750 vs CAE_750 | 0.64 |  |  |  |
| -> direct |  | -0.295 | -0.692 | 0.089 |
| -> indirect |  | -0.324 | -0.493 | -0.15 |
| -> MBNMA |  | -0.318 | -0.476 | -0.156 |
| RT_250 vs CAE_500 | 0.385 |  |  |  |
| -> direct |  | -0.006 | -0.327 | 0.319 |
| -> indirect |  | 0.021 | -0.054 | 0.098 |
| -> MBNMA |  | 0.021 | -0.052 | 0.093 |
| HIIT_750 vs CAE_500 | 0.565 |  |  |  |
| -> direct |  | -0.336 | -0.764 | 0.08 |
| -> indirect |  | -0.414 | -0.585 | -0.24 |
| -> MBNMA |  | -0.401 | -0.559 | -0.242 |
| HIIT_250 vs CAE_500 | 0.137 |  |  |  |
| -> direct |  | -0.179 | -1.108 | 0.725 |
| -> indirect |  | -0.025 | -0.084 | 0.038 |
| -> MBNMA |  | -0.025 | -0.085 | 0.038 |
| CE_1000 vs CAE_500 | 0.523 |  |  |  |
| -> direct |  | -0.142 | -0.642 | 0.346 |
| -> indirect |  | -0.2 | -0.372 | -0.018 |
| -> MBNMA |  | -0.193 | -0.365 | -0.025 |
| Yoga_1000 vs Placebo_0 | 0.549 |  |  |  |
| -> direct |  | -0.591 | -1.03 | -0.161 |
| -> indirect |  | -0.316 | -0.782 | 0.146 |
| -> MBNMA |  | -0.467 | -0.793 | -0.153 |
| Yoga_500 vs Placebo_0 | 0.556 |  |  |  |
| -> direct |  | -0.226 | -0.692 | 0.237 |
| -> indirect |  | -0.228 | -0.404 | -0.06 |
| -> MBNMA |  | -0.233 | -0.397 | -0.077 |
| TCS_1500 vs Placebo_0 | 0.252 |  |  |  |
| -> direct |  | -0.381 | -0.782 | 0.011 |
| -> indirect |  | -0.742 | -0.966 | -0.528 |
| -> MBNMA |  | -0.656 | -0.857 | -0.467 |
| TCS_1250 vs Placebo_0 | 0.006 |  |  |  |
| -> direct |  | -1.244 | -1.647 | -0.835 |
| -> indirect |  | -0.44 | -0.607 | -0.271 |
| -> MBNMA |  | -0.547 | -0.714 | -0.389 |
| TCS_1000 vs Placebo_0 | 0.412 |  |  |  |
| -> direct |  | -0.568 | -0.852 | -0.289 |
| -> indirect |  | -0.407 | -0.548 | -0.256 |
| -> MBNMA |  | -0.437 | -0.571 | -0.311 |
| TCS_500 vs Placebo_0 | 0.415 |  |  |  |
| -> direct |  | -0.159 | -0.384 | 0.055 |
| -> indirect |  | -0.229 | -0.295 | -0.16 |
| -> MBNMA |  | -0.219 | -0.286 | -0.156 |
| RT_750 vs Placebo_0 | 0.552 |  |  |  |
| -> direct |  | -0.303 | -0.719 | 0.122 |
| -> indirect |  | -0.492 | -0.758 | -0.228 |
| -> MBNMA |  | -0.428 | -0.636 | -0.211 |
| HIIT_500 vs Placebo_0 | 0.411 |  |  |  |
| -> direct |  | -0.275 | -0.483 | -0.055 |
| -> indirect |  | -0.412 | -0.538 | -0.292 |
| -> MBNMA |  | -0.375 | -0.486 | -0.263 |
| CE_750 vs Placebo_0 | 0.391 |  |  |  |
| -> direct |  | -0.116 | -0.526 | 0.267 |
| -> indirect |  | -0.28 | -0.416 | -0.148 |
| -> MBNMA |  | -0.267 | -0.399 | -0.137 |
| CE_500 vs Placebo_0 | 0.586 |  |  |  |
| -> direct |  | -0.169 | -0.414 | 0.067 |
| -> indirect |  | -0.18 | -0.274 | -0.091 |
| -> MBNMA |  | -0.178 | -0.266 | -0.092 |
| CAE_1500 vs Placebo_0 | 0.353 |  |  |  |
| -> direct |  | -0.302 | -0.687 | 0.085 |
| -> indirect |  | -0.52 | -0.681 | -0.369 |
| -> MBNMA |  | -0.488 | -0.629 | -0.339 |
| CAE_1250 vs Placebo_0 | 0.709 |  |  |  |
| -> direct |  | -0.382 | -0.608 | -0.138 |
| -> indirect |  | -0.415 | -0.554 | -0.282 |
| -> MBNMA |  | -0.407 | -0.524 | -0.282 |
| CAE_250 vs Placebo_0 | 0.098 |  |  |  |
| -> direct |  | 0.051 | -0.415 | 0.507 |
| -> indirect |  | -0.082 | -0.105 | -0.058 |
| -> MBNMA |  | -0.081 | -0.105 | -0.056 |

**Table S16.** Node-splitting method of network transitivity for LDL-C

| **Comparison** | **p-value** | **Median** | **2.50%** | **97.50%** |
| --- | --- | --- | --- | --- |
| TCS_750 vs CAE_1000 | 0.437 |  |  |  |
| -> direct |  | 0.267 | -0.317 | 0.84 |
| -> indirect |  | 0.181 | 0.022 | 0.343 |
| -> MBNMA |  | 0.19 | 0.034 | 0.342 |
| HIIT_1000 vs CAE_1000 | 0.64 |  |  |  |
| -> direct |  | -0.159 | -0.516 | 0.194 |
| -> indirect |  | -0.011 | -0.309 | 0.283 |
| -> MBNMA |  | -0.071 | -0.296 | 0.166 |
| CE_1000 vs CAE_1000 | 0.372 |  |  |  |
| -> direct |  | 0.117 | -0.387 | 0.631 |
| -> indirect |  | -0.156 | -0.369 | 0.039 |
| -> MBNMA |  | -0.112 | -0.306 | 0.063 |
| Yoga_750 vs CAE_750 | 0.755 |  |  |  |
| -> direct |  | -0.277 | -0.73 | 0.179 |
| -> indirect |  | -0.125 | -0.719 | 0.469 |
| -> MBNMA |  | -0.223 | -0.569 | 0.125 |
| RT_500 vs CAE_750 | 0.654 |  |  |  |
| -> direct |  | 0.055 | -0.18 | 0.3 |
| -> indirect |  | -0.038 | -0.233 | 0.161 |
| -> MBNMA |  | 0.001 | -0.161 | 0.16 |
| RT_250 vs CAE_750 | 0.168 |  |  |  |
| -> direct |  | -0.232 | -0.884 | 0.484 |
| -> indirect |  | 0.172 | 0.07 | 0.28 |
| -> MBNMA |  | 0.163 | 0.062 | 0.267 |
| HIIT_750 vs CAE_750 | 0.589 |  |  |  |
| -> direct |  | -0.099 | -0.558 | 0.356 |
| -> indirect |  | -0.044 | -0.226 | 0.137 |
| -> MBNMA |  | -0.053 | -0.222 | 0.124 |
| RT_250 vs CAE_500 | 0.474 |  |  |  |
| -> direct |  | 0.034 | -0.253 | 0.324 |
| -> indirect |  | 0.063 | -0.026 | 0.152 |
| -> MBNMA |  | 0.054 | -0.03 | 0.14 |
| HIIT_750 vs CAE_500 | 0.6 |  |  |  |
| -> direct |  | -0.138 | -0.56 | 0.321 |
| -> indirect |  | -0.141 | -0.328 | 0.044 |
| -> MBNMA |  | -0.161 | -0.325 | 0.01 |
| HIIT_250 vs CAE_500 | 0.106 |  |  |  |
| -> direct |  | 0.147 | -1.228 | 1.591 |
| -> indirect |  | 0.091 | 0.026 | 0.16 |
| -> MBNMA |  | 0.091 | 0.024 | 0.162 |
| CE_1000 vs CAE_500 | 0.093 |  |  |  |
| -> direct |  | 0.147 | -0.337 | 0.619 |
| -> indirect |  | -0.395 | -0.585 | -0.216 |
| -> MBNMA |  | -0.332 | -0.509 | -0.165 |
| Yoga_500 vs Placebo_0 | 0.707 |  |  |  |
| -> direct |  | -0.275 | -0.69 | 0.154 |
| -> indirect |  | -0.401 | -0.704 | -0.096 |
| -> MBNMA |  | -0.367 | -0.597 | -0.135 |
| TCS_1500 vs Placebo_0 | 0.191 |  |  |  |
| -> direct |  | -0.189 | -0.59 | 0.19 |
| -> indirect |  | -0.608 | -0.838 | -0.359 |
| -> MBNMA |  | -0.49 | -0.704 | -0.286 |
| TCS_1250 vs Placebo_0 | 0.466 |  |  |  |
| -> direct |  | -0.574 | -1.044 | -0.136 |
| -> indirect |  | -0.379 | -0.571 | -0.188 |
| -> MBNMA |  | -0.408 | -0.586 | -0.238 |
| TCS_1000 vs Placebo_0 | 0.676 |  |  |  |
| -> direct |  | -0.357 | -0.658 | -0.053 |
| -> indirect |  | -0.321 | -0.491 | -0.162 |
| -> MBNMA |  | -0.327 | -0.469 | -0.19 |
| TCS_500 vs Placebo_0 | 0.431 |  |  |  |
| -> direct |  | -0.24 | -0.479 | 0.003 |
| -> indirect |  | -0.161 | -0.236 | -0.083 |
| -> MBNMA |  | -0.163 | -0.235 | -0.095 |
| RT_750 vs Placebo_0 | 0.863 |  |  |  |
| -> direct |  | -0.481 | -0.869 | -0.091 |
| -> indirect |  | -0.511 | -0.824 | -0.226 |
| -> MBNMA |  | -0.485 | -0.731 | -0.252 |
| HIIT_500 vs Placebo_0 | 0.543 |  |  |  |
| -> direct |  | -0.159 | -0.406 | 0.1 |
| -> indirect |  | -0.265 | -0.398 | -0.125 |
| -> MBNMA |  | -0.252 | -0.371 | -0.128 |
| CE_1250 vs Placebo_0 | 0.002 |  |  |  |
| -> direct |  | -1.548 | -2.04 | -1.092 |
| -> indirect |  | -0.461 | -0.677 | -0.253 |
| -> MBNMA |  | -0.686 | -0.915 | -0.465 |
| CE_750 vs Placebo_0 | 0.195 |  |  |  |
| -> direct |  | -0.798 | -1.406 | -0.184 |
| -> indirect |  | -0.391 | -0.537 | -0.264 |
| -> MBNMA |  | -0.412 | -0.549 | -0.279 |
| CE_500 vs Placebo_0 | 0.324 |  |  |  |
| -> direct |  | -0.141 | -0.405 | 0.128 |
| -> indirect |  | -0.292 | -0.386 | -0.204 |
| -> MBNMA |  | -0.274 | -0.366 | -0.186 |
| CAE_1250 vs Placebo_0 | 0.657 |  |  |  |
| -> direct |  | -0.478 | -0.765 | -0.15 |
| -> indirect |  | -0.562 | -0.749 | -0.385 |
| -> MBNMA |  | -0.544 | -0.693 | -0.399 |

***Note:*** The value is expressed as the specific exercise modality followed by corresponding exercise

dose of that modality. Placebo: control group; CAE, continuous aerobic exercise; CE, combined aerobic and resistance exercise; RT, resistance training; TCS, traditional Chinese sports.

**Figure S10.** Density plot showing the distribution of direct and indirect effects from node-splitting method (a, TG; b, LDL-C)

a

b

***Note:*** Title in each plot indicates the specific exercise modality followed by corresponding exercise

dose of that modality. Placebo: control group; CAE, continuous aerobic exercise; CE, combined aerobic and resistance exercise; RT, resistance training; TCS, traditional Chinese sports.

**8.2 Model selection**

8.2.1 Examination on the relationship between weekly exercise dose and blood lipid levels

A “split” NMA of different weekly exercise dose was performed at the overall (all exercise

modalities being lumped together) and agent levels. This step helps determine which function is more appropriate to be applied in the model-based dose-response NMA.

**Figure S11.** "Split" NMA showing the effects of weekly exercise dose on TG (a, overall; b, agent-level) and LDL-C (c, overall; d, agent-level)

a	b

c	d

***Note:*** Effect sizes are presented in MD with 95% CrI. CAE, continuous aerobic exercise; CE, combined aerobic and resistance exercise; RT, resistance training; TCS, traditional Chinese sports.

8.2.2 Non-linear functions and model fit comparisons

For our data, the quadratic random-effects model showed the best fit for both TG and LDL-C.

**Table S17.** Model fit comparisons

| TG | | | | | |
| --- | --- | --- | --- | --- | --- |
| Model | DIC | SD | Deviance | Residual deviance | pD |
| Emax (common treatment effects) | 56.8 | NA | -17.176 | 284.679 | 74.1 |
| Restricted cubic spline (common treatment effects; 3 knots) | -20.3 | NA | -109.627 | 192.228 | 89.3 |
| Restricted cubic spline (random treatment effects; 3 knots) | -43.1 | 0.139  (0.08,0.20) | -154.122 | 147.733 | 110.0 |
| Nonmonotonic model (common treatment effects) | 343.2 | NA | 274.151 | 576.006 | 69.4 |
| Quadratic (2^nd^ degree polynomial, common treatment effects) | -7.4 | NA | -87.032 | 214.823 | 80.1 |
| Quadratic (2^nd^ degree polynomial, random treatment effects) | -45.4 | 0.159  (0.11,0.22) | -152.380 | 149.475 | 107.8 |
| LDL-C | | | | | |
| Model | DIC | SD | Deviance | Residual deviance | pD |
| Emax (common treatment effects) | 24.0 | NA | -42.986 | 254.643 | 67.1 |
| Restricted cubic spline (common treatment effects; 3 knots) | -31.0 | NA | -112.057 | 185.572 | 81.3 |
| Restricted cubic spline (random treatment effects; 3 knots) | -45.2 | 0.144  (0.04,0.24) | -146.756 | 150.873 | 103.3 |
| Nonmonotonic model (common treatment effects) | 282.2 | NA | 220.419 | 518.048 | 62.1 |
| Quadratic (2nd degree  polynomial, common treatment effects) | -7.5 | NA | -80.259 | 217.371 | 72.9 |
| Quadratic (2nd degree polynomial, random treatment effects) | -48.2 | 0.187  (0.12,0.27) | -152.252 | 145.377 | 103.8 |

In addition to the model fit indices, a deviance plot showing the contribution of each data point to the residuals helps to confirm the robustness of model selection. The contribution of each data point to the posterior mean around 1.0 indicates good model fit.

**Figure S12.** Deviance plots for TG (a, overall; b, agent-level) and LDL-C (c, overall; d, agent-level)

a

b

c

d

***Note:*** CAE, continuous aerobic exercise; CE, combined aerobic and resistance exercise; RT, resistance training; TCS, traditional Chinese sports.

Fit plots are drawn to further assess the degree of model fit. The fit values are plotted as connecting lines, and the observations in the original dataset are plotted as points. These plots can help determine whether the model fits data well for different exercise modalities and weekly exercise dose from the dose-response function.

**Figure S13.** Fit plots for TG (a, overall; b, agent-level) and LDL-C (c, overall; d, agent-level)

a

b

c

d

***Note:*** CAE, continuous aerobic exercise; CE, combined aerobic and resistance exercise; RT, resistance training; TCS, traditional Chinese sports.

**8.3 Dose-response relationships**

8.3.1 Dose-response relationships between weekly exercise dose and blood lipid levels

a	b

c	d

**Figure S14.** Dose-response relationship between weekly exercise dose and TG

***Note:*** The left panel shows the dose-response relationship at the overall level and the right at the agent level. The upper panel represents the dose-response relationship along with “split” NMA

results, and the lower panel represents the dose-response relationship along with the distribution of the original dataset shaded in green color (the darker the color, the larger the amount of data). CAE, continuous aerobic exercise; CE, combined aerobic and resistance exercise; RT, resistance training; TCS, traditional Chinese sports.

]

a	b

c	d

**Figure S15.** Dose-response relationship between weekly exercise dose and LDL-C

***Note:*** The left panel shows the dose-response relationship at the overall level and the right at the agent level. The upper panel represents the dose-response relationship along with “split” NMA

results, and the lower panel represents the dose-response relationship along with the distribution of the original dataset shaded in green color (the darker the color, the larger the amount of data). CAE, continuous aerobic exercise; CE, combined aerobic and resistance exercise; RT, resistance training; TCS, traditional Chinese sports.

8.3.2 Ranking the effectiveness of different exercise modalities combined with dose on blood lipid levels

**Figure S16.** Effectiveness ranking of exercise modality combined with weekly exercise dose on TG

***Note:*** Title in each plot indicates the specific exercise modality followed by corresponding weekly exercise dose of that modality. Placebo: control group; CAE, continuous aerobic exercise; CE, combined aerobic and resistance exercise; RT, resistance training; TCS, traditional Chinese sports.

**Table S18.** Predictions ranking for TG (from best to worst)

| **Treatment** | **Mean** | **Median** | **2.50%** | **97.50%** |
| --- | --- | --- | --- | --- |
| HIIT_1000 | 1.4 | 1 | 1 | 4 |
| HIIT_890 | 3.02 | 3 | 2 | 6 |
| TCS_1500 | 3.29 | 3 | 1 | 10.03 |
| TCS_1300 | 5.38 | 5 | 2 | 12 |
| HIIT_780 | 5.64 | 5 | 3 | 11 |
| TCS_1200 | 7.31 | 7 | 4 | 14 |
| Yoga_1000 | 9.75 | 6 | 1 | 41 |
| HIIT_670 | 10.34 | 9 | 5 | 21 |
| TCS_1000 | 11.68 | 11 | 7 | 22 |
| Yoga_890 | 13.38 | 10 | 4 | 36 |
| CE_1000 | 15.98 | 14 | 6 | 34 |
| CAE_1300 | 17.17 | 16 | 8 | 34 |
| CAE_1200 | 17.32 | 16 | 10 | 30 |
| CAE_1500 | 18.37 | 15 | 4 | 44.03 |
| TCS_830 | 18.52 | 18 | 9 | 32 |
| CAE_1000 | 19.11 | 19 | 12 | 28 |
| HIIT_560 | 19.15 | 18 | 8 | 36 |
| Yoga_780 | 19.76 | 17 | 6 | 42 |
| CE_890 | 20.37 | 20 | 9 | 34 |
| RT_580 | 21.31 | 21 | 10 | 36 |
| RT_500 | 21.81 | 22 | 10 | 35 |
| RT_670 | 22.49 | 21 | 7 | 44 |
| CAE_830 | 22.59 | 22 | 14 | 32 |
| RT_420 | 23.86 | 24 | 10 | 38 |
| RT_750 | 24.77 | 23 | 4 | 55 |
| CE_780 | 25.61 | 26 | 12 | 39 |
| TCS_670 | 26.66 | 27 | 13 | 40 |
| CAE_670 | 26.99 | 27 | 17 | 37 |
| Yoga_670 | 27.27 | 27 | 7.98 | 50 |
| RT_330 | 27.91 | 28 | 12 | 42 |
| HIIT_440 | 30.28 | 31 | 13 | 46 |
| CE_670 | 30.61 | 31 | 13 | 46 |
| CAE_500 | 32.49 | 33 | 23 | 42 |
| RT_250 | 32.65 | 33 | 16.98 | 47 |
| Yoga_560 | 33.6 | 35 | 9 | 52 |
| TCS_500 | 34.74 | 35 | 21 | 46 |
| CE_560 | 35.23 | 36 | 16 | 51 |
| RT_170 | 38.25 | 38 | 26 | 50 |
| CAE_330 | 38.5 | 38.5 | 30 | 47 |
| HIIT_330 | 38.79 | 39 | 22 | 53 |
| Yoga_440 | 39.2 | 41 | 12 | 54 |
| CE_440 | 39.68 | 40 | 21 | 53 |
| TCS_330 | 41.66 | 42 | 31 | 51 |
| CE_330 | 43.57 | 44 | 27 | 55 |
| Yoga_330 | 43.7 | 46 | 16 | 55 |
| CAE_170 | 44.9 | 45 | 38 | 51.03 |
| RT_83 | 45.04 | 45 | 36 | 53 |
| HIIT_220 | 45.15 | 46 | 32 | 55 |
| CE_220 | 47.06 | 48 | 35 | 55 |
| TCS_170 | 47.39 | 48 | 39 | 54 |
| Yoga_220 | 47.41 | 50 | 27 | 55 |
| HIIT_110 | 49.71 | 50 | 41 | 55 |
| CE_110 | 50 | 50 | 42 | 55 |
| Yoga_110 | 50.29 | 52 | 39 | 55 |
| Placebo_0 | 51.9 | 52 | 45 | 55 |

***Note:*** Each treatment is presented as the specific exercise modality followed by corresponding

weekly exercise dose of that modality. Placebo: control group; CAE, continuous aerobic exercise; CE, combined aerobic and resistance exercise; RT, resistance training; TCS, traditional Chinese sports.

**Figure S17.** Effectiveness ranking of exercise modality combined with weekly exercise dose on LDL-C

***Note:*** Title in each plot indicates the specific exercise modality followed by corresponding weekly exercise dose of that modality. Placebo: control group; CAE, continuous aerobic exercise; CE, combined aerobic and resistance exercise; RT, resistance training; TCS, traditional Chinese sports.

**Table S19.** Predictions ranking for LDL-C (from best to worst)

| **Treatment** | **Mean** | **Median** | **2.50%** | **97.50%** |
| --- | --- | --- | --- | --- |
| CE_1200 | 1.32 | 1 | 1 | 4 |
| CE_1100 | 3.03 | 2 | 2 | 7 |
| HIIT_1000 | 6.88 | 4 | 1 | 27 |
| CE_970 | 7.63 | 7 | 3 | 18 |
| Yoga_750 | 7.8 | 4 | 1 | 37 |
| HIIT_890 | 10.15 | 8 | 3 | 27 |
| CAE_1200 | 10.22 | 8 | 3 | 30 |
| Yoga_670 | 10.38 | 7 | 3 | 32 |
| CAE_1100 | 11.21 | 10 | 5 | 25 |
| CAE_970 | 13.38 | 13 | 7 | 23 |
| HIIT_780 | 15.8 | 15 | 6 | 32 |
| Yoga_580 | 16.66 | 14 | 4 | 41 |
| CAE_830 | 17.14 | 17 | 9 | 27 |
| RT_580 | 18.72 | 18 | 7 | 34 |
| RT_670 | 18.84 | 17 | 5 | 41 |
| CE_830 | 20.04 | 20 | 6 | 36.03 |
| RT_750 | 20.2 | 17 | 2.98 | 50 |
| RT_500 | 20.26 | 20 | 8 | 34 |
| TCS_1200 | 20.65 | 20 | 7 | 37 |
| TCS_1000 | 20.72 | 21 | 8 | 34 |
| TCS_1300 | 21.59 | 21 | 6 | 42 |
| CAE_690 | 22.16 | 22 | 12 | 33 |
| TCS_830 | 22.85 | 23 | 10 | 36 |
| HIIT_670 | 23.1 | 23 | 8 | 39 |
| RT_420 | 23.19 | 23 | 9 | 37 |
| Yoga_500 | 23.26 | 22 | 4 | 50 |
| TCS_1500 | 24.79 | 24 | 3 | 53 |
| TCS_670 | 26.59 | 27 | 12 | 39 |
| CAE_560 | 27.5 | 28 | 15 | 38 |
| RT_330 | 27.81 | 28 | 12 | 42 |
| Yoga_420 | 29.22 | 30 | 4 | 52 |
| HIIT_560 | 30.1 | 31 | 12 | 46 |
| TCS_500 | 31.74 | 32 | 18 | 44 |
| RT_250 | 32.9 | 33 | 18 | 46 |
| CAE_420 | 33.42 | 34 | 22 | 43 |
| CE_690 | 33.5 | 34 | 13 | 49 |
| Yoga_330 | 34.93 | 37 | 6 | 54 |
| HIIT_440 | 36.57 | 37 | 17 | 51 |
| TCS_330 | 37.65 | 38 | 26 | 48 |
| RT_170 | 38.5 | 39 | 26 | 49 |
| CAE_280 | 39.45 | 40 | 31 | 48 |
| Yoga_250 | 39.75 | 42 | 10 | 55 |
| HIIT_330 | 41.66 | 42 | 26 | 54 |
| CE_560 | 42.04 | 43 | 23 | 52 |
| TCS_170 | 43.99 | 44 | 36 | 51 |
| Yoga_170 | 44.2 | 47 | 19 | 55 |
| RT_83 | 45.04 | 45 | 37 | 52 |
| CAE_140 | 45.56 | 46 | 39 | 52 |
| HIIT_220 | 45.96 | 46 | 34 | 55 |
| CE_420 | 47.72 | 49 | 32 | 54 |
| Yoga_83 | 48.44 | 50 | 36 | 55 |
| HIIT_110 | 49.27 | 50 | 42 | 55 |
| Placebo_0 | 51.03 | 51 | 45 | 55 |
| CE_280 | 51.21 | 53 | 40 | 55 |
| CE_140 | 52.26 | 53 | 46 | 55 |

***Note:*** Each treatment is presented as the specific exercise modality followed by corresponding weekly exercise dose of that modality. Placebo: control group; CAE, continuous aerobic exercise; CE, combined aerobic and resistance exercise; RT, resistance training; TCS, traditional Chinese sports.
